# Aberrant Metabolic Patterns Networks in Insular Epilepsy

**DOI:** 10.3389/fneur.2020.605256

**Published:** 2020-12-23

**Authors:** Baotian Zhao, Caio Seguin, Lin Ai, Tao Sun, Wenhan Hu, Chao Zhang, Xiu Wang, Chang Liu, Yao Wang, Jiajie Mo, Andrew Zalesky, Kai Zhang, Jianguo Zhang

**Affiliations:** ^1^Department of Neurosurgery, Beijing Tiantan Hospital, Capital Medical University, Beijing, China; ^2^China National Clinical Research Center for Neurological Diseases, Beijing, China; ^3^Melbourne Neuropsychiatry Centre, The University of Melbourne and Melbourne Health, Melbourne, VIC, Australia; ^4^Department of Imaging and Nuclear Medicine, Beijing Tiantan Hospital, Capital Medical University, Beijing, China; ^5^Department of Neurosurgery, General Hospital of Ningxia Medical University, Yinchuan, China; ^6^Department of Biomedical Engineering, Melbourne School of Engineering, The University of Melbourne, Melbourne, VIC, Australia

**Keywords:** insular epilepsy, metabolic covariance network, neocortical, subcortical, brainstem

## Abstract

**Introduction:** Insular epilepsy is clinically challenging. This study aimed to map cerebral metabolic networks in insular epilepsy and investigate their graph-theoretic properties, with the goal of elucidating altered metabolic network architectures that underlie interictal hypometabolism.

**Aims:** Fluorine-18-fluorodeoxyglucose positron emission tomography (^18^F-FDG-PET) imaging was performed in 17 individuals with a stereoelectroencephalography (SEEG) confirmed diagnosis of insula epilepsy and 14 age- and sex-matched healthy comparison individuals. Metabolic covariance networks were mapped for each group and graph theoretical analyses of these networks were undertaken. For each pair of regions comprising a whole-brain parcellation, regionally-averaged FDG uptake values were correlated across individuals to estimate connection weights.

**Results:** Correlation in regionally-averaged FDG uptake values in the insular epilepsy group was substantially increased for several pairs of regions compared to the healthy comparison group, particularly for the opercular cortex and subcortical structures. This effect was less prominent in brainstem structures. Metabolic covariance networks in the epilepsy group showed reduced small-worldness as well as altered nodal properties in the ipsilateral hemisphere, compared to the healthy comparison group.

**Conclusions:** Cerebral glucose metabolism in insular epilepsy is marked by a lack of normal regional heterogeneity in metabolic patterns, resulting in metabolic covariance networks that are more tightly coupled between regions than healthy comparison individuals. Metabolic networks in insular epilepsy exhibit altered topological properties and evidence of potentially compensatory formation of aberrant local connections. Taken together, these results demonstrate that insular epilepsy is a systemic neurological disorder with widespread disruption to cerebral metabolic networks.

## Highlights

- Insular epilepsy is associated with interictal disturbances in metabolic coupling between the insula and neocortical-subcortical-brainstem structures.
- Metabolic brain networks in insular epilepsy show aberrant topological organization, characterized by an excess of local connections.
- Metabolic brain networks in insular epilepsy show high local synchronization, possibly resulting from epileptic discharge and inefficient global communication.

## Introduction

The insula (or insular cortex) is functionally and structurally connected to a diverse array of cortical and subcortical areas, and serves to integrate information across multiple functional networks (1). The diversity of ictal clinical symptoms observed in insular epilepsy is therefore consistent with the diversity of neocortical and subcortical targets connected to this hub region (1–3). Seizure semiology of insular seizures is far from being homogeneous and present with various seizure manifestations, including gustatory/auditory auras, somatosensory, autonomic symptoms (vomiting, piloerection, heart rate changes), and motor manifestations (automotor, hypermotor, tonic, clonic behaviors) (4). Insular seizures have several clinical patterns depending on the anatomical brain regions of seizure spread, therefore supporting insular epilepsy is a great mimicker (5). This renders the clinical recognition of insular epilepsy arduous (6). Presurgical evaluations, including scalp electroencephalography (EEG) and neuroimaging, often fail to precisely pinpoint the epileptogenic zone, eventually requiring invasive EEG (iEEG) monitoring to confirm the location of the epileptic focus (7). Hence, insular epilepsy remains clinically challenging. Surgical iEEG implantation is often considered for individuals with drug-resistant insular epilepsy; however, this requires knowledge of the approximate location of the epileptogenic zone and its interactions with other epileptic networks. Therefore, improved techniques are needed to map these networks pre-surgically and thereby characterize the epileptogenic zone with greater fidelity.

Fluorine-18-fluorodeoxyglucose positron emission tomography (^18^F-FDG-PET) is an established neuroimaging marker of glucose metabolism and has been widely used to localize regions of hypometabolism in individuals with epilepsy, often referred to as functional deficit zones (8). Hypometabolism may be related to ictal onset and seizure propagation to distant regions (9). Metabolic covariance networks provide a principled approach to investigate metabolic effects across a spatially distributed set of regions. Given that the insula is a hub region, and that insular epilepsy is associated with distributed metabolic effects that extend beyond the epileptogenic zone (10), metabolic covariance networks are a promising candidate to understand these brain-wide effects. In these networks, nodes correspond to gray matter regions and connections are drawn between pairs of nodes for which metabolic rates (e.g., FDG uptake) correlate across a group of individuals. In this way, regions are connected if they share comparable metabolic rates. Metabolic covariance networks for glucose metabolism have been investigated in healthy adults as well as individuals with neuropsychiatric disorders and temporal lobe epilepsy (11, 12). These studies demonstrate the damaging effects and disrupted internetwork connectivity in temporal lobe epilepsy patients. However, few studies have investigated metabolic networks in insular epilepsy (13).

Epileptic seizures involve widespread network interactions among neocortical, subcortical, and brainstem structures (14, 15). Focal epilepsy is increasingly considered a disease of abnormal brain network organization and function, affecting large-scale brain networks beyond the epileptogenic zones (16). Ictal seizure events may lead to persistent interictal neocortical/subcortical network disturbances that may affect both brain connectivity and neurocognitive function. Previous studies identified that many of the subcortical regions are most critical for neocortical activation brainstem structures (ascending reticular activating system, ARAS) for arousal (15, 17).

The aim of the present study was to investigate brain networks in insular epilepsy from a metabolic standpoint. We hypothesized that interictal hypometabolism could recapitulate networks involved in epileptic discharges. Metabolic covariance network was constructed to explore whether the aberrant topology of metabolic networks in insular epilepsy could co-localize with regions that generate ictal onset and spread. And the graph-theoretical analysis was used to investigate metabolic covariance networks in individuals with insular epilepsy.

## Methods

### Demographic

Seventeen individuals (seven females) with SEEG confirmed insular epilepsy were recruited in this study during the evaluation for iEEG and epilepsy surgery at Beijing Tiantan Hospital from 2015 to 2018. Criteria for inclusion in this study included the following: (1) surgical resection to treat focal epilepsy; (2) high quality of imaging data (without motion artifact, aliasing or rippling related to eye movement) and SEEG recordings (without noise); (3) no history of dystocia hypoxia, encephalitis or severe traumatic brain injury; (4) no encephalomalacia and severe or diffuse brain atrophy; and (5) non-reoperation. In addition, 14 healthy individuals (five females) were recruited to match the mean age and sex ratio of the epileptic group. Exclusion criteria for the control group included any history of psychiatric or neurological disorders.

The site of the epileptogenic focus was confirmed with presurgical evaluation, such as the MRI/PET and iEEG findings. Seizure outcomes was rated for all patients using ILAE classification: (1) Class 1: completely seizure free without auras; (2) Class 2: only auras, no other seizures; (3) Class 3: 1–3 seizure days/year, ± auras; (4) Class 4: four seizure days/year to 50% reduction of baseline seizure days, ± auras; (5) Class 5: <50% reduction of baseline seizure days to 100% increase of baseline seizure days; ± auras; (6) more than 100% increase of baseline seizure days; ± auras (18). Informed consent was obtained from all individuals, and this study was approved by the Ethics Board of the Beijing Tiantan Hospital, Capital Medical University.

### Neuroimaging Data Acquisition and Pre-processing

#### Neuroimaging Data Acquisition

MRI scans were performed on a 3T Siemens Verio scanner including a 3D T1 sagittal magnetization prepared rapid gradient echo sequence (MPRAGE; TR = 1,900 milliseconds, TE = 2.53 milliseconds, TI = 900 milliseconds, flip angle = 12°, slice thickness = 1 mm, no gap, matrix = 256 × 256, voxel size = 0.98 × 0.98 × 1 mm^3^).

PET scans of all patients were obtained in the interictal state with the same protocols as healthy subjects. The ^18^FDG-PET examinations were performed under standard resting conditions using the GE Discovery ST PET-CT system (300 mm FOV, matrix 192 × 192, 3.27 mm slice thickness). Patients were required to rest quietly in a dimly lit room during the 40 min following 18F-FDG intravenous administration of a mean dose of 310 MBq/70 kg body weight. Ordered subset expectation maximization (OSEM) algorithm (16 subsets and six iterations) was used for PET data reconstruction. The reconstructed images were corrected for attenuation using transmission scans obtained from a germanium source. PET scans of all patients were obtained within 6 months before epilepsy surgery evaluation. No patients had clinical seizures <6 h before or during the PET scan.

#### Neuroimaging Pre-processing

**Figure 1:**
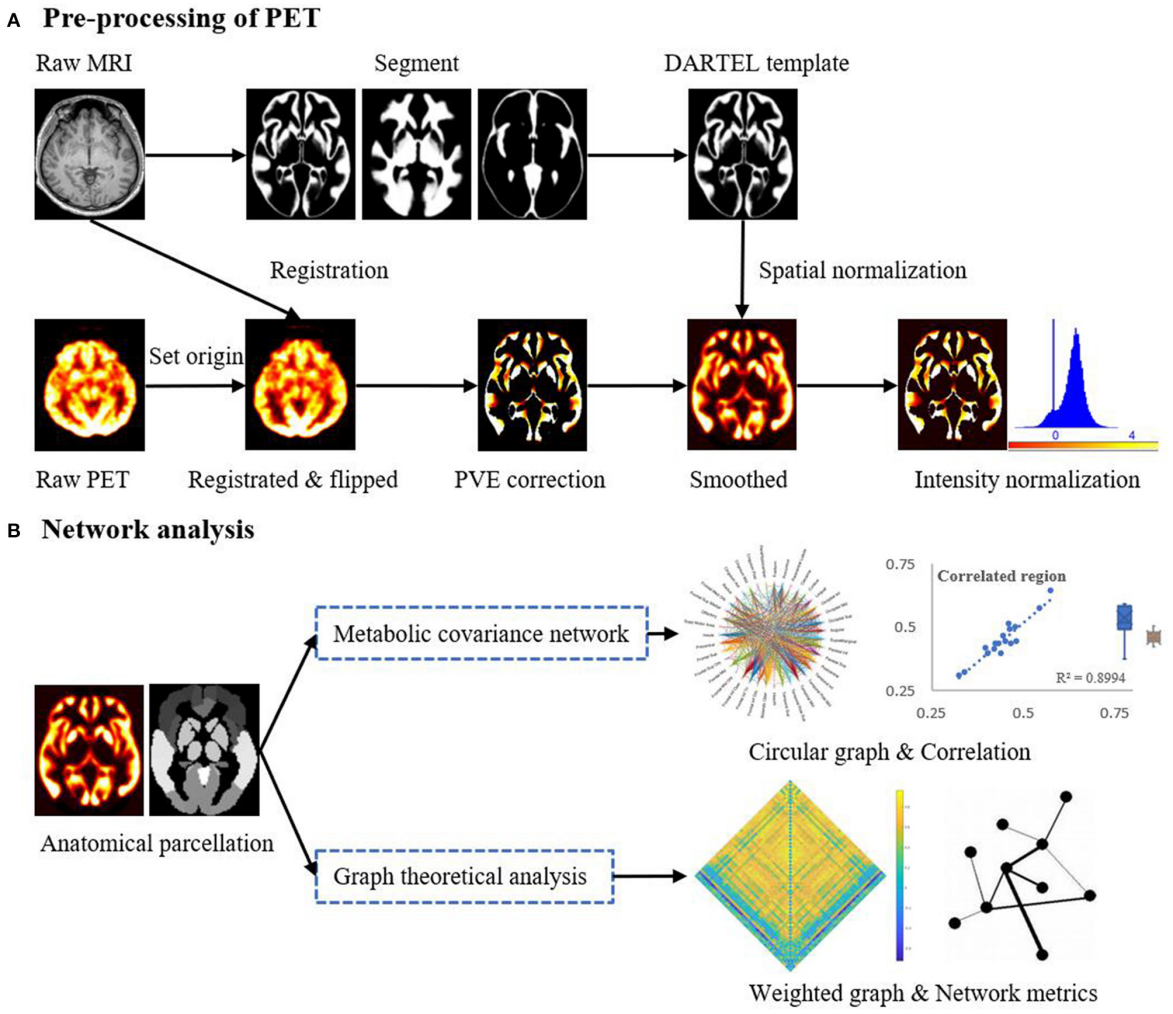
Schematic representation of the steps in **(A)** the pre-processing of the PET imaging data and **(B)** mapping of metabolic covariance networks. The topological properties of metabolic covariance networks were compared using graph-theoretical measurements between epileptic and healthy individuals. DARTEL, diffeomorphic anatomical registration through exponentiated lie algebra; MRI, magnetic resonance imaging; PET, positron emission tomography; PVE, partial volume effect.

Figure 1 provides a schematic of the pre-processing steps for the ^18^F-FDG-PET images and subsequent network analysis. Analysis of the ^18^F-FDG-PET images was performed in MATLAB 2018a (The MathWorks, Natick, Massachusetts, USA) using SPM12 (Wellcome Department of Cognitive Neurology, University College, London, UK) and PETPVE12 software (https://github.com/GGonEsc/petpve12) (19). In brief, the images were processed as follows: (1) Setting the origin. The center of mass of the PET images was approximately corrected to lie on the anterior commissure. (2) Registration. PET images were co-registered with individual magnetic resonance images, and data quality was examined. (3) Flipping. Horizontal PET images of patients with right epileptogenic foci were side-flipped using SPM's image calculator “ImCalc” with the required expression “flipud (i1),” which enabled the analysis to be performed uniformly and increased the sample size and sensitivity of the statistical analyses. (4) Creation of the Diffeomorphic Anatomical Registration Through Exponentiated Lie Algebra (DARTEL) template (20). Parameter files produced by the segmentation were used to interactively compute a group-specific template. (5) Partial volume effects (PVE) correction. The modified MG method (21) used segmented tissue compartments (gray matter [GM], white matter [WM], and cerebrospinal fluid [CSF]) to correct the PET GM signal for spill-in effects from surrounding tissue, typically WM signals. (6) Spatial normalization and smoothing. High-dimensional DARTEL normalization was used to normalize the PET images to a generative template according to normalization parameters. The non-linear modulated DARTEL normalized images were then smoothed with a 5 mm isotropic Gaussian kernel. (7) Intensity normalization. The PET signal was standardized utilizing a reference value (cerebellar GM) to enable inter-subject comparison. The normalized PET signal (FDG uptake) was used in all subsequent analyses.

### Metabolic Covariance Networks

Normalized FDG uptake values were averaged across voxels, each comprised of a number of distinct regions, thereby yielding a single uptake value for each region in each individual. Regions were selected from the Automated Anatomical Labeling (AAL) atlas (http://www.gin.cnrs.fr/en/tools/aal-aal2/) and Harvard Ascending Arousal Network (AAN) atlas (martinos.org/resources/aan-atlas). Given that epileptogenic foci in the right hemisphere were flipped to the left as part of the pre-processing pipeline, all regions residing in the right hemisphere were excluded. Cerebellar regions were also excluded. In total, 53 regions (39 neocortical, six subcortical, and eight brainstem) that served as network nodes were selected (Supplementary Table 2). For each pair of nodes, FDG uptake values were correlated across the group of epileptic individuals, while controlling for the effect of FDG uptake values at all remaining nodes. This resulted in a 53 × 53 symmetric correlation matrix, in which each matrix element stored a partial correlation coefficient. Large correlation strengths in this matrix indicated pairs of nodes that shared similar metabolic rates across individuals (i.e., covarying metabolic rates across individuals). The same process was repeated for the group of healthy individuals. It is important to note that metabolic covariance networks are group-level representations and can only be mapped for a group of individuals, but not for any particular individual. Abbreviations and the full names of the included brain structures are displayed in Supplementary Table 2.

### Network Analysis

Analysis of the metabolic covariance networks was foremost constrained to the insula. The correlation strengths between the insula and each of the other 52 regions were considered. Connections were drawn between the insula and other regions for which the correlation strength survived Bonferroni correction (*P* < 0.05). Negative correlations were omitted. The set of positive connections surviving Bonferroni correction were then compared between the epileptic and healthy groups.

In addition to focussing on the insula, graph-theoretical analyses were employed to investigate network-wide effects in the metabolic covariance networks. To this end, global and nodal topological characteristic of these networks were investigated and benchmarked to randomized networks (22). In particular, the small-worldness, global efficiency, hierarchy, and synchronization were computed for the epileptic and healthy groups. At the nodal level, the clustering coefficient, degree centrality, local efficiency, and betweenness centrality were analyzed. These properties were computed using the GRaphthEoreTical Network Analysis (GRETNA) toolbox (23) and are described in further detail in the Supplementary Material. Each of these measurements were computed for a range of connection densities (5–50%, with 5% increments), and the area under the curve (AUC) was used to provide a single summary measure across this range. Thresholding was performed such that the weakest connections according to the correlation coefficient were successively eliminated until the prescribed connection density was reached. Statistical inference was performed on the AUC for each measurement. The Maslov-Sneppen rewiring algorithm was used to generate an ensemble of reference networks that were matched in connection density, number of nodes, and node degree to the actual metabolic covariance networks (24). The above-described topological characteristics were computed in the reference networks, and the resulting values were used to normalize the values in the actual networks.

### Statistical Analysis

Demographic and clinical data were analyzed using SPSS (version 20.0, SPSS Inc., Chicago, IL, USA). A two-sample *t*-test and a chi-squared test were used to compare continuous and categorical clinical variables and graph-theoretical properties, respectively, between the two groups. The statistical significance threshold was set at *P* < 0.05. The Pearson correlation coefficient was utilized to examine metabolic connectivity between the insula and other cortical regions. Corrections for multiple comparisons were performed using the Bonferroni-Holm method where applicable.

## Results

### Clinical Characteristics

The mean (± standard deviation [SD]) age was 16.25 ± 9.85 years for the individuals with insular epilepsy and 22.64 ± 7.55 years for the healthy individuals. The duration of epilepsy was 9.27 ± 8.09 years. Clinical characteristics, including semiology, neuroimaging findings, SEEG results, surgical resection, post-operative pathology and prognosis are provided in Table 1. Epileptic foci were located in the left hemisphere in 10 (58.8%) individuals. Gyri lesions were similar in quantity and extent among individuals, with four (23.5%) individuals exhibiting widespread lesions involving multiple gyri. Nine resections exhibited a histopathological diagnosis of malformation of cortical dysplasia, and five demonstrated focal neocortical gliosis.

**Table 1:** Demographic characteristics of individuals with insular epilepsy.

**Patient**	**Age/Sex (yrs)**	**Semiology**	**MRI findings**	**PET findings**	**Epileptogenic focus by SEEG**	**Surgery**	**Histology**	**Follow-up (months)**	**Outcome**
1	M/17	Dyspnoea aura; FT (R face)-R dystonia-BATS-Bil eye blinking and perioral clonic drooling	L insular “transmantle sign”	L insular hypometabolism	L MSG	L Frontal Inf Tri, insula	Not specific	59	ILAE 1
2	M/5.5	Nod-Bil arm tonic-FT (Rt face)	L insular cortical thickening	L insular hypometabolism	L ASG	L Frontal Inf Tri, insula	FCD I	58	ILAE 1
3	M/32	L body numbness aura; FT (neck, face)-eyes blinking, R arm tonic and hand dystonia-L arm tonic	NA	NA	R ALG	R Parietal Inf, insula	Not specific	55	ILAE 3
4	M/30	L body undescribed sensation aura; L arm tonic-FT (R face)-grimace-R HMS	R insular signal abnormality	NA	R ALG	R Parietal Inf, PSG, ALG, PLG	FCD IIa	48	ILAE 1
5	M/22	FT (neck, L face)-eyes R deviation-BATS (R arm flexion)	L insular signal abnormality	NA	L MSG	L Ronlandic Oper, MSG	FCD IIa	48	ILAE 3
6	M/13	R arm undescribed sensation aura; FT (L face)-R arm tonic-HMS	NA	NA	L PSG	L PSG, ALG, MLG	Not specific	48	ILAE 2
7	F/10	Epigastric-numbness of whole-body aura; FT (neck, L face)-R arm tonic, L hand rhythmic shaking	NA	L temporal and insular hypometabolism	R MSG	R Frontal Inf Tri, ASG, MSG, PSG	FCD IIa	42	ILAE 1
8	F/13	R arm undescribed sensation aura; FT (neck)-R dystonic-L dystonic, FT (L face)	NA	NA	L PSG	L Frontal Oper, MSG, PSG, ALG, PLG	FCD IIa	41	ILAE 1
9	M/14	Cephalic sensation aura; FT (neck, R face)-eyes R deviation, Bil arm tonic-L eye blinking	L insular cortical thickening	L insular and temporal pole hypometabolism	L PSG, ALG, MLG	L Frontal Oper, insula	Not specific	35	ILAE 1
10	F/3.3	R clonic-eye blinking	NA	L ALG and PLG hypometabolism	L ALG, PLG	L Frontal Oper, ALG, PLG	FCD IIa	39	ILAE 3
11	F/8	Fear aura; Blinking-L arm tonic-Bil hand automatism	NA	L temporal, parietal and insular hypometabolism	L superior temporal gyrus, ALG, PLG	L Heschl gyrus, ALG, PLG	FCD IIa	37	ILAE 1
12	F/33	eye blinking-swallowing-R arm tonic-FT (R face)	L insular signal abnormality	L temporal and insular hypometabolism	L PSG	L PSG, ALG	Not specific	37	ILAE 1
13	M/27	L face and arm numbness aura; Increased heart rate, L arm clonic, Drooling	R insular opercular signal abnormality	R insular opercular hypometabolism	R parietal operculum, insula	R Parietal Oper, MSG, PSG, ALG, PLG	Not specific	28	ILAE 3
14	M/7	Shrug-R arm tonic-FT (L face)	NA	R insular opercular hypometabolism	R ALG	R PSG, ALG	FCD IIb	30	ILAE 1
15	M/9	L arm numbness aura; FT (neck)-eyes R deviation-Bil arm tonic and R hand dystonia-eyes blinking and FC (R face)-Bilateral legs HMS-Drooling	R insular opercular signal abnormality	R insular opercular hypometabolism	R MSG, PSG, ALG	R Frontal Oper, MSG, PSG, ALG	FCD IIa	30	ILAE 1
16	F/24	FT (R face, neck)-Drooling, R arm tonic and hand dystonia-L hand dystonia- trunk twisting left and right	NA	L insular hypometabolism	L PSG, ALG, PLG	L Frontal Oper, PSG, ALG, PLG	Not specific	32	ILAE 1
17	F/7	Staring-FT (L face)	NA	R Frontal Orb, insular hypometabolism	R PSG, ALG, PLG	R Temporal Oper, PSG, ALG, PLG	Not specific	31	ILAE 1

*FT, focal tonic seizure; BATS, bilateral asymmetric tonic seizure; HMS, hypermotor seizure; L, left side; R, right side; Bil, bilateral side; ASG, anterior short gyrus; MSG, middle short gyrus; PSG, posterior short gyrus; ALG, anterior long gyrus; PLG, posterior long gyrus; NA, not specific*.

### Insular Metabolic Covariance

**Figure 2:**
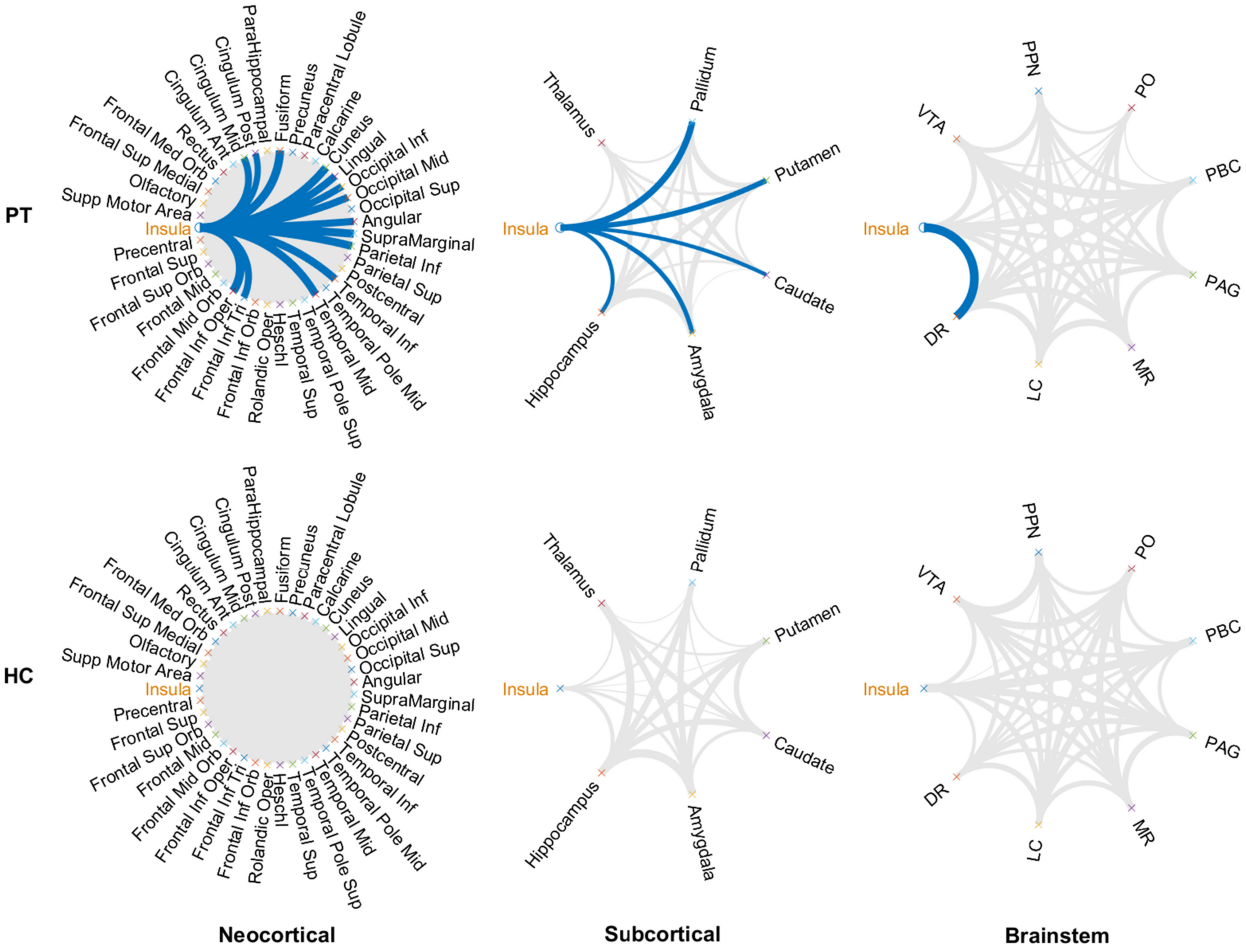
Insula-to-cortex metabolic covariance network for a group of individuals with insular epilepsy (upper panel) and a group of healthy individuals (lower panel). Networks stratified according to neocortical (left), subcortical (center), and brainstem (right) regions. Line thickness is modulated by the correlation coefficient, and connections that survive correction for multiple comparisons across the set of all connections are indicated in blue (*P* < 0.05, Bonferroni corrected). While FDG uptake values in the epileptic group were significantly correlated between the insula and numerous regions of the neocortical, subcortical, and brainstem structures, no such associations were evident in the healthy group. DR, dorsal raphé; LC, locus coeruleus; MR, median raphe; PAG, periaqueductal gray; PBC, parabrachial complex; PO, pontis oralis; PPN, pedunculopontine nucleus; VTA, ventral tegmental area.

**Figure 3:**
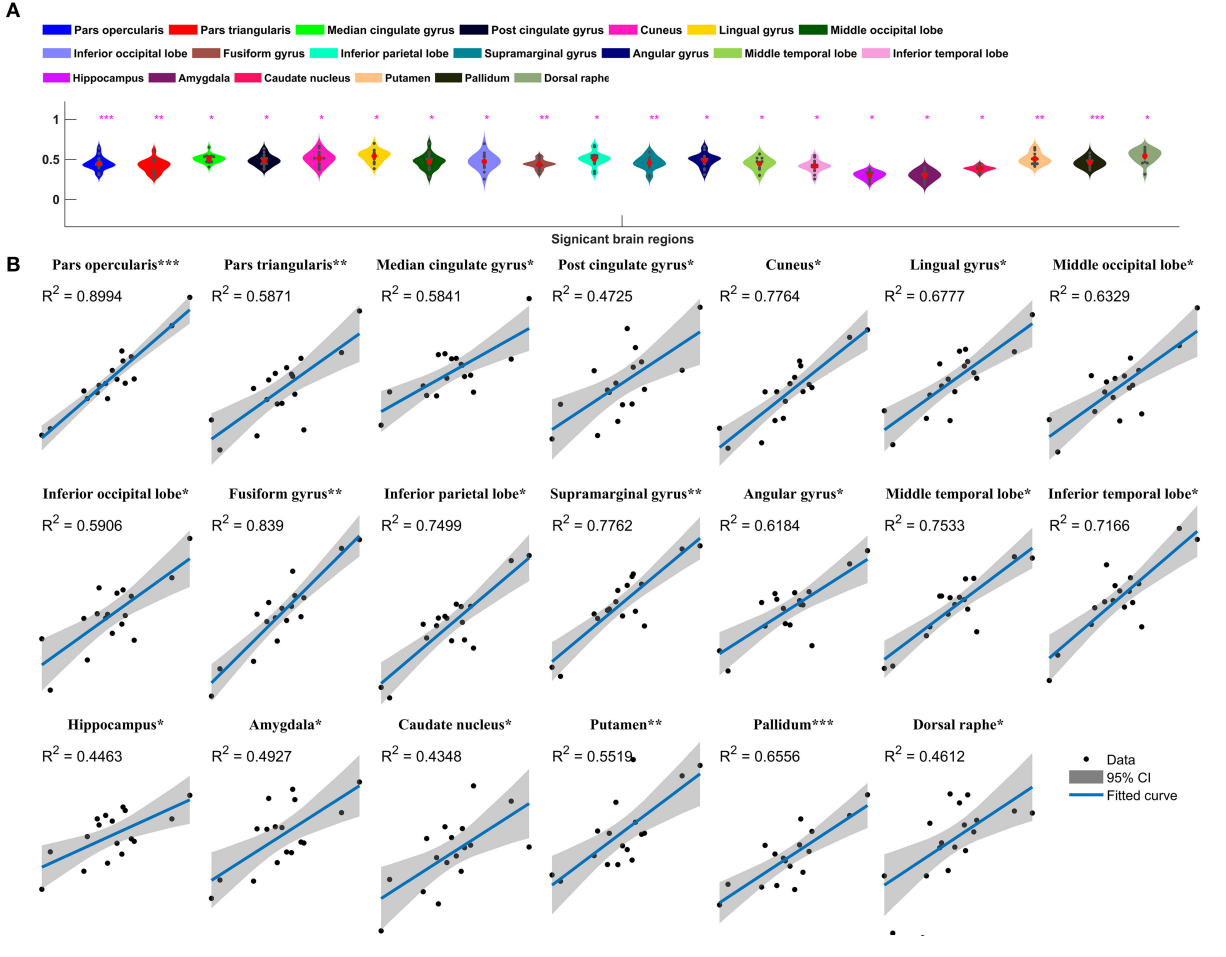
Insula-to-cortex metabolic covariance network in a group of individuals with insular epilepsy. **(A)** Boxplots showing FDG uptake values (*P* < 0.05, Bonferroni corrected). Solid red circles denote mean values and gray dots denote individuals. **(B)** Scatter plots showing the covariance across epileptic individuals in FDG uptake values between the insula and other regions. Solid black circles denote individuals. The horizontal axis shows normalized FDG uptake values for the insula, the vertical axis corresponds to the region indicated in the title. For each scatter plot, the blue line denotes the line of best fit, and shading denotes the 95% confidence interval. *P < 0.05, **P < 0.01, ***P < 0.001.

Metabolic covariance was measured between the insula and 39 neocortical, six subcortical, and eight brainstem regions in the epileptic and healthy group. The metabolic covariance between the insula and a given region was quantified as the correlation in FDG uptake values across individuals. Compared to the healthy individuals, epileptic individuals showed significantly greater metabolic covariance between the insula and the following neocortical and subcortical regions: pars opercularis, pars triangularis, middle temporal lobe, inferior temporal lobe, inferior parietal lobe, supramarginal gyrus, angular gyrus, middle occipital lobe, inferior occipital lobe, lingual gyrus, cuneus, fusiform gyrus, median cingulate gyrus, and post-cingulate gyrus at the neocortical level; the hippocampus, amygdala, caudate nucleus, putamen, and pallidum at the subcortical level; and the dorsal raphé nucleus at the brainstem level. In the control group, FDG uptake values in the insula were not significantly correlated with uptake values in any other regions (Bonferroni corrected, *P* < 0.05). Figure 2 shows a circular representation of the insula-to-cortex metabolic network for the epileptic (upper panel) and healthy group (lower panel). Figure 3 shows scatter plots that exemplify the covariance across the epileptic individuals in FDG uptake values between the insula and other regions.

### Regional Between-Group Differences in FDG Uptake

**Figure 4:**
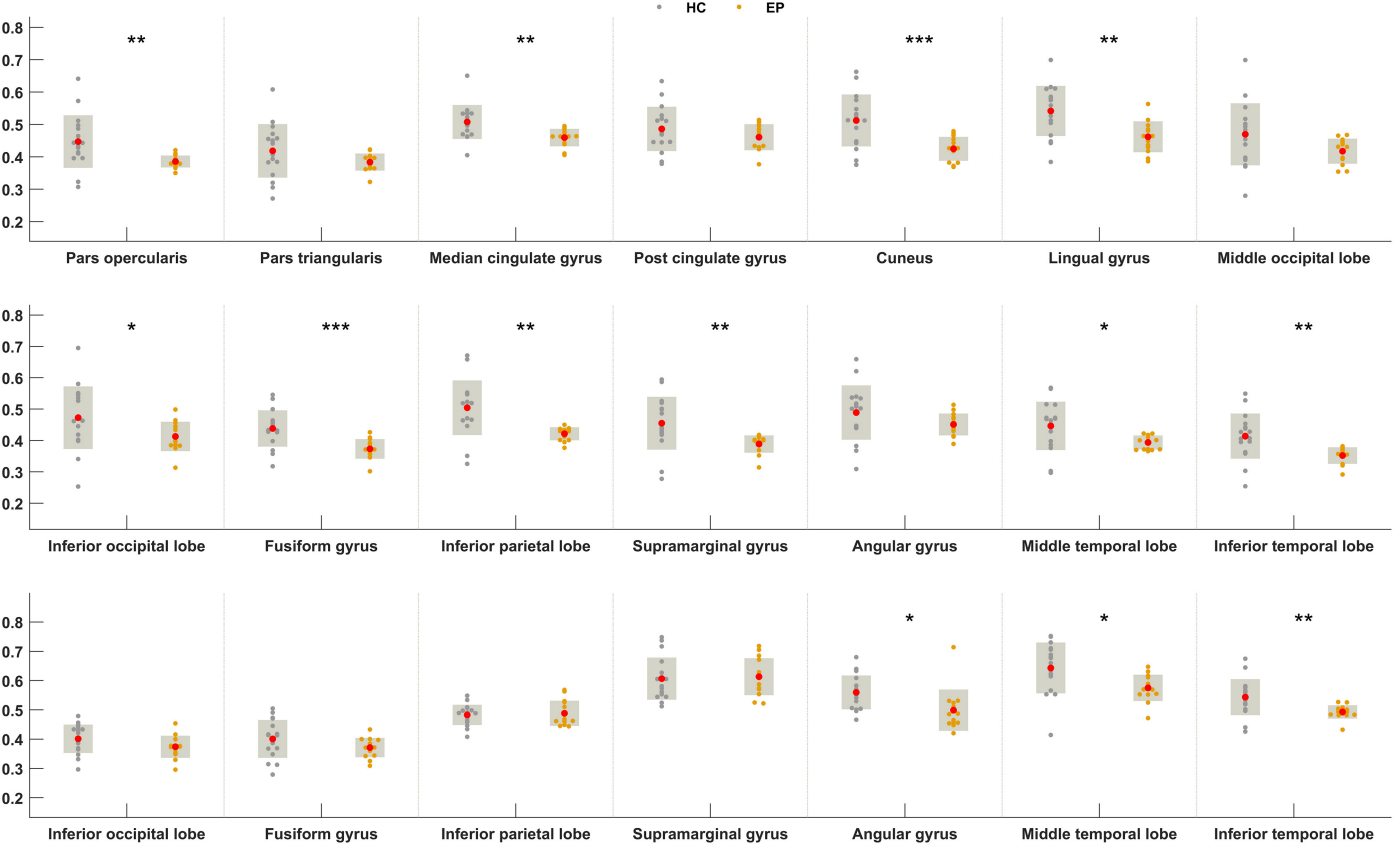
Between-group differences in FDG uptake for regions showing greater metabolic covariance with the insula. Orange and gray circles denote FDG uptake values in individuals with insular epilepsy and healthy individuals, respectively. Solid red circles denote the mean FDG uptake. HC, Healthy group; EP, Epileptic group. *P < 0.05, **P < 0.01, ***P < 0.001.

We next sought to establish whether the increased metabolic covariance associated with epilepsy was accompanied by regional alterations in glucose metabolism, as indexed by FDG uptake. Between-group differences were only tested at the regions found to show significant metabolic covariance with the insula in the epileptic group (see above). Relative to the healthy group, epilepsy was associated with significantly lower FDG uptake (hypometabolism) in numerous regions, including the insula, pars opercularis, median cingulate gyrus, cuneus, lingual gyrus, inferior occipital lobe, fusiform gyrus, inferior parietal lobe, supramarginal gyrus, middle temporal lobe, and inferior temporal lobe at the neocortical level. For the subcortical and brainstem structures, hypometabolism was confined to the pallidum and dorsal raphé, respectively. Figure 4 shows boxplots exemplifying these regional between-group differences in FDG uptake.

### Topological Properties of Metabolic Networks

**Figure 5:**
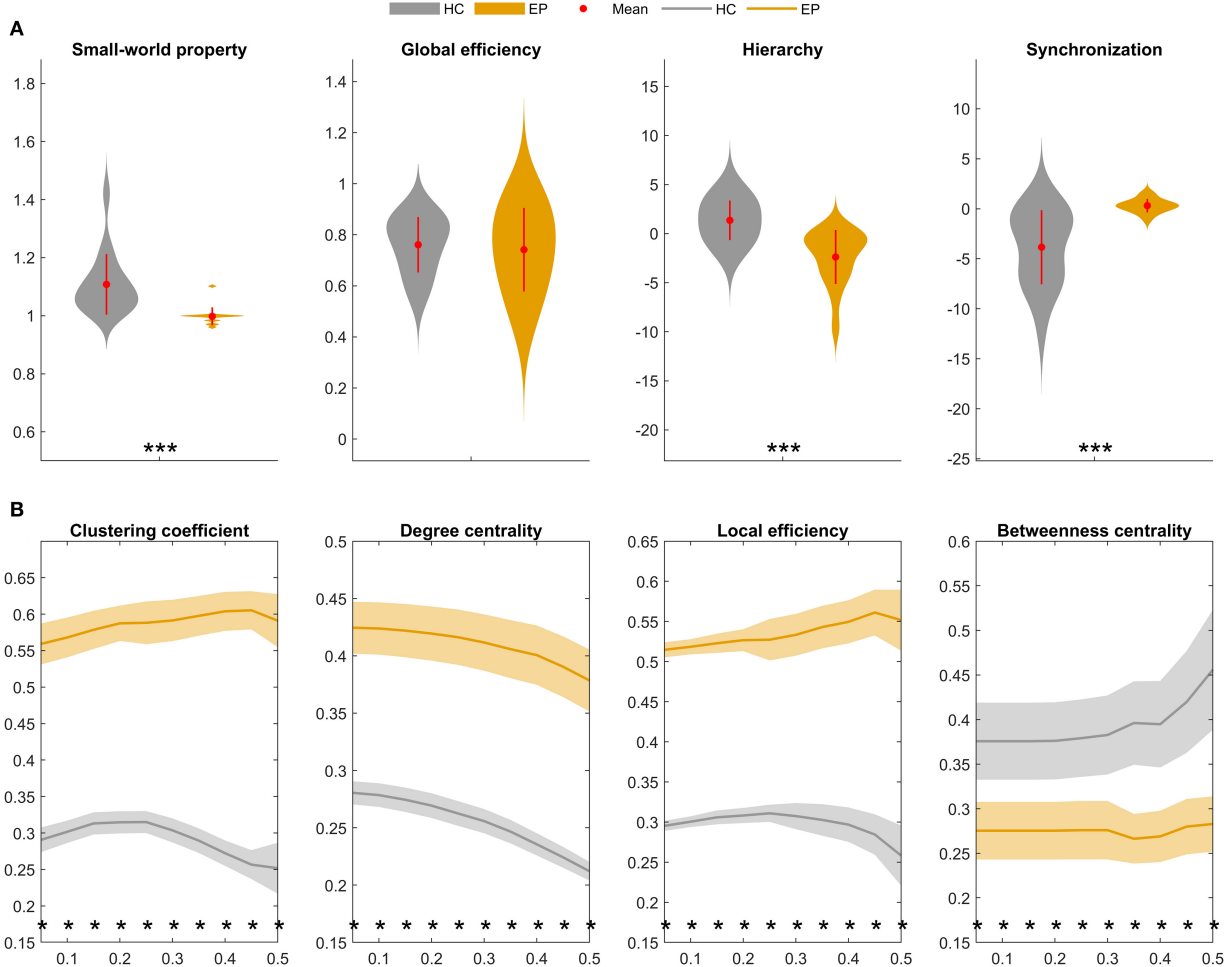
Global and local topological properties of metabolic covariance networks compared between individuals with insular epilepsy (orange) and healthy individuals (gray). **(A)** Between-group differences in global brain network properties displaying group means (red circles), group standard deviations (red lines), and stars (significant between-group differences). Compared to the healthy group, small-worldness (sigma, σ) was significantly reduced in the epileptic group (*P* < 0.01*), whereas global efficiency was not significantly different (*P* = 0.223). **(B)** Nodal brain network properties as a function of sparsity threshold (5–50%, with steps of 5%). Stars denote significant between-group differences at the respective sparsity value. The line and shading denote the mean and 95% CI of between-group differences, respectively, at each sparsity value. HC, Healthy group; EP, Epileptic group. *P < 0.05, **P < 0.01, ***P < 0.001.

Finally, graph-theoretical analyses were performed on the whole-brain metabolic networks mapped for the epileptic and healthy groups. A small-world network is considered as a short characteristic path length between individual regions and a high degree of clustering, represented a key topological feature in healthy brain network. Moreover, the global topological properties represent long-range connecting efficiency within the nodes of brain network, which can be employed to measure the ability of information communication of the network at global levels. Global efficiency measures the global efficiency of parallel information transfer in a network. Synchronization measures correlated alteration and fluctuation in all nodes. The hierarchy coefficient is used to identify the presence of a hierarchical organization in a network. Local topological properties are mainly combined to short-range connections among neighboring regions to mediate modularized information processing or fault tolerance of a network. The clustering coefficient of a given node measures the likelihood its neighborhoods are connected to each other. The nodal degree for a given node reflects its information communication ability in the network. The local efficiency for a given node measures how efficient the communication is among the first neighbors of this node when it is removed. The nodal betweenness for a given node characterizes its effect on communication between other nodes (23, 25). Figure 5 compares summary statistics of the network measurements investigated in this study.

Global topological properties of the metabolic networks were investigated first (Figure 5A). Summary measurements (area under the curve) of the small-worldness (*P* < 0.05), hierarchy (*P* < 0.05), and global efficiency (*P* > 0.05) parameters were lower in the epileptic group compared to the healthy group, while synchronization (*P* < 0.05) was higher in the epileptic group.

Nodal network properties (Figure 5B) were significantly higher in the epileptic group, including the clustering coefficient (C), degree centrality (DC), and local efficiency (Eloc), with significant between-group differences (*P* < 0.05) for sparsity ranging between 5 and 50%. However, in terms of betweenness centrality (BC), the epileptic group showed smaller values than the healthy group and the between-group differences (*P* < 0.05) were significant for sparsity ranging between 5 and 50%. Taken together, our findings suggest that metabolic brain networks in insular epilepsy are characterized by reduced small-worldness, together with significant regional increases in clustering, degree centrality, and local efficiency, compared to healthy individuals.

## Discussion

^18^F-FDG-PET plays a crucial role in the presurgical evaluation of intractable epilepsy. In this study, we used this neuroimaging modality to map a metabolic covariance network in a group of individuals with insular epilepsy. Connections in the metabolic network represented pairs of neocortical, subcortical, and/or brainstem regions for which FDG uptake values significantly covaried across the group of epileptic individuals. We mapped the same kind of metabolic covariance network for a group of healthy individuals and compared connectivity strength and topological properties between the two groups. We found significant metabolic covariance in the epileptic group between the insula and numerous regions of the cortical, subcortical, and brainstem structures, whereas the healthy group showed no evidence of metabolic covariance between these regions. Moreover, the metabolic covariance network mapped in the epileptic group showed markedly different local and global topological properties compared to the network mapped for the healthy group.

### Brain Metabolism and Covariance Networks

Clinically, hypometabolism in the brain that is detected with ^18^F-FDG-PET is usually associated with the occurrence and propagation of epileptic discharge, especially synchronous metabolic changes in the cortex (9). Interictal PET hypometabolism had a significant correlation with ictal high-frequency oscillations (iHFO) confirmed by SEEG, which indicated that they may share common underlying pathophysiologic mechanisms of ictogenesis (26). The insula is anatomically and functionally interconnected with widespread adjacent perisylvian and distant neocortical regions, which has been confirmed by previous cortico-cortical evoked potentials (CCEP) and tractography studies (5, 27). Sustained seizure activity abnormally increases excitability, which may produce cell loss and reduce glucose intake in both seizure-generating regions and areas of seizure spread (28). Given these considerations, we contend that the metabolic covariance in FDG uptake observed in the present study may represent a marker of seizure propagation in epileptic networks.

In particular, we suggest that metabolic covariance could be interpreted in the context of metabolic patterns of insular epilepsy. Seizure semiology mostly relies on epileptic network organization, including location of the epileptogenic zone and propagation networks (29). This study identified the interictal hypometabolic network, including the pars opercularis and pars triangularis. Dense connections between the insular cortex and the operculum underlie the insulo-opercular network and can be presumed to play a role in the frequently observed insulo-opercular hypometabolic pattern, which is concordant with previous study confirmed by SEEG ictal signals (30). Compared to temporal lobe epilepsy and frontal lobe epilepsy, parietal lobe seizures are by far the least studied (31). Both structures of the inferior parietal lobe and parietal operculum (comprising the supramarginal and angular gyrus) have been shown to participate in the modulation of sensory processing (32). Parietal-opercular regions have privileged connections with the premotor cortex in humans (33), which contribute to the anatomical basis for prominent motor manifestations (particularly hyperkinetic behavior), similar to the anterior operculo-insular cortex. The posterior cingulate gyrus and associative parietal cortex are involved in the processing of normal consciousness (34). In contrast to the epileptic group, we did not find any pairs of regions that showed statistically significant levels of metabolic covariance in the healthy group. This further suggests that metabolic covariance specifically indexes the consequences of a pathological process, possibly marking out the route of seizure spread within the epilepsy network. The putative route of seizure spread included several extra cortical regions, such as the occipital lobe, cuneus, and lingual gyrus. Epileptogenic zones in these areas are seldom reported in the existing literature and typically neglected given that they do not map to clinical symptoms. If metabolic networks do indeed mark the route of seizure spread, our results indicate the downstream consequences of seizure are evident at most cortical and subcortical regions.

As a subcortical structure that has widespread interactions with almost all cortical regions (1), the basal ganglia have been described as a physiologic synchronizer of seizures (35). Recurrent seizures may lead to abnormal connectivity patterns involving subcortical structures important for cortical activation, leading in turn to pathological neocortical connectivity and function (36). While metabolic covariance should not be confused with measurements of structural and functional connectivity, it is possible that metabolic covariance may potentially recapitulate patterns of abnormal connectivity in epilepsy. We noted that the dorsal raphé nucleus, the largest serotonergic nucleus, was the only brainstem region with significant metabolic covariance with the insula. The abnormal metabolic pattern of the dorsal raphé nucleus suggests that it may be associated with autonomic dysfunction and motor symptoms (head movements, rigidity patterns) during seizure and neuropsychological impairment.

### Topological Properties of Metabolic Networks

The metabolic network mapped for the healthy group showed small-world organization, combining strong local connectivity and efficient long-distance connections, which enables both the specialization and integration of distributed networks while reducing wiring costs and facilitating information flow (37). Our findings demonstrate a pathological pattern of network organization in insular epilepsy, which was characterized by low global efficiency, hierarchy, and betweenness centrality, as well as high synchronization, clustering, degree centrality, and local efficiency. In line with previous studies, decreased global efficiency of information transfer in temporal lobe epilepsy and an increase in the clustering coefficient may result from the compensatory formation of aberrant local connections in response to a decrease in the number of long-range connections. In addition, this more regularized network has been reported to deteriorate over time, becoming susceptible to targeted attacks and associated with poor postsurgical seizure outcome (38).

### Limitations and Further Considerations

Our findings should be interpreted with consideration to several limitations. First, the number of individuals comprising the epileptic group was modest. However, insular epilepsy is rare, and we adopted strict inclusion criteria, including simultaneous diagnosis by neuroimaging data and stereoelectroencephalography (SEEG). Metabolic covariance networks were mapped at the group level as covariance was measured across a group of individuals, and thus it was not possible to undertake individual inference. Future work should consider investigating whether aberrant network patterns of metabolic covariance in insular epilepsy recapitulate disruptions in functional and structural connectivity that can be mapped with EEG and MRI, and whether these patterns carry prognostic utility. It will also be important to ascertain whether successful surgical treatment or antiepileptic medication can reverse the aberrant patterns of metabolic covariance found in this study.

## Conclusions

Our results provide preliminary evidence suggesting that insular epilepsy is a systemic neurological disease that is characterized by abnormal metabolic covariance not only involving the insular cortex, but a widespread network spanning the neocortical, subcortical, and brainstem structures. These distributed effects most likely contribute to the diverse clinical manifestations of insular epilepsy. Furthermore, metabolic networks in insular epilepsy show marked reorganization with respect to global and local topology of metabolic covariance networks.

## Data Availability Statement

The original contributions generated for this study are included in the article/Supplementary Material, further inquiries can be directed to the corresponding author/s.

## Ethics Statement

The studies involving human participants were reviewed and approved by the Ethics Board of the Beijing Tiantan Hospital, Capital Medical University. Written informed consent to participate in this study was provided by the participants' legal guardian/next of kin.

## Author Contributions

JZ, BZ, and AZ conceived and planned the study. JZ and KZ performed the surgery resection, discussed the results, and revised the manuscript. CS performed the statistical analysis and drafted the manuscript. TS, CZ, and XW worked out almost all the technical details. YW and CL collected the data. JM did the figures. All authors provided critical feedback and helped shape the research, analysis and the manuscript.

## Conflict of Interest

The authors declare that the research was conducted in the absence of any commercial or financial relationships that could be construed as a potential conflict of interest.

## References

[1] Tian Y Zalesky A. Characterizing the functional connectivity diversity of the insula cortex: subregions, diversity curves and behavior. NeuroImage. (2018) 183:716–33. 10.1016/jneuroimage201808055 30172005

[2] Cauda F Costa T Torta DM Sacco K D'Agata F Duca S. Meta-analytic clustering of the insular cortex: characterizing the meta-analytic connectivity of the insula when involved in active tasks. NeuroImage. (2012) 62:343–55. 10.1016/jneuroimage201204012 22521480 PMC4782788

[3] Ghaziri J Tucholka A Girard G Houde JC Boucher O Gilbert G. The corticocortical structural connectivity of the human insula. Cereb Cortex. (2017) 27:1216–28. 10.1093/cercor/bhv308 26683170

[4] Jobst BC Gonzalez-Martinez J Isnard J Kahane P Lacuey N Lahtoo SD. The insula and Its epilepsies. Epilepsy Curr. (2019) 19:11–21. 10.1177/1535759718822847 30838920 PMC6610377

[5] Laoprasert P Ojemann JG Handler MH. Insular epilepsy surgery. Epilepsia. (2017) 58:35–45. 10.1111/epi13682 28386920

[6] Haller M Case J Crone NE Chang EF King-Stephens D Laxer KD. Persistent neuronal activity in human prefrontal cortex links perception and action. Nat Hum Behav. (2017) 2:80–91. 10.1038/s41562-017-0267-2 29963646 PMC6022844

[7] Gras-Combe G Minotti L Hoffmann D Krainik A Kahane P Chabardes S. Surgery for nontumoral insular epilepsy explored by stereoelectroencephalography. Neurosurgery. (2016) 79:578–88. 10.1227/NEU0000000000001257 27244467

[8] Goffin K Dedeurwaerdere S Van L Aere K Van Paesschen W. Neuronuclear assessment of patients with epilepsy. Semin Nucl Med. (2008) 38:227–39. 10.1053/jsemnuclmed200802004 18514079

[9] Chassoux F Semah F Bouilleret V Landre E Devaux B Turak B. Metabolic changes and electro-clinical patterns in mesio-temporal lobe epilepsy: a correlative study. Brain. (2004) 127:164–74. 10.1093/brain/awh014 14534161

[10] Wang X Hu W Zhang K Shao X Ma Y Sang L. The anatomo-electrical network underlying hypermotor seizures. Front Neurol. (2018) 9:243. 10.3389/fneur201800243 29695997 PMC5904199

[11] Lee DS Kang H Kim H Park H Oh JS Lee JS. Metabolic connectivity by interregional correlation analysis using statistical parametric mapping (SPM) and FDG brain PET; methodological development and patterns of metabolic connectivity in adults. Eur J Nucl Med Mol Imaging. (2008) 35:1681–91. 10.1007/s00259-008-0808-z 18491089

[12] Nugent AC Martinez A D'Alfonso A Zarate CA Theodore WH. The relationship between glucose metabolism, resting-state fMRI BOLD signal, and GABAA-binding potential: a preliminary study in healthy subjects and those with temporal lobe epilepsy. J Cereb Blood Flow Metab. (2015) 35:583–91. 10.1038/jcbfm2014228 25564232 PMC4420874

[13] Bouilleret V Dupont S Spelle L Baulac M Samson Y Semah F. Insular cortex involvement in mesiotemporal lobe epilepsy: a positron emission tomography study. Ann Neurol. (2002) 51:202–8. 10.1002/ana10087 11835376

[14] Norden AD Blumenfeld H. The role of subcortical structures in human epilepsy. Epilepsy Behav. (2002) 3:219–31. 10.1016/S1525-5050(02)00029-X 12662601

[15] Englot DJ Gonzalez HFJ Reynolds BB Konrad PE Jacobs ML Gore JC. Relating structural and functional brainstem connectivity to disease measures in epilepsy. Neurology. (2018) 91:e67–77. 10.1212/WNL0000000000005733 29848786 PMC6091881

[16] Richardson MP. Large scale brain models of epilepsy: dynamics meets connectomics. J Neurol Neurosurg Psychiatry. (2012) 83:1238–48. 10.1136/jnnp-2011-301944 22917671

[17] Výtvarová E Mareček R Fousek J Strýček O Rektor I. Large-scale cortico-subcortical functional networks in focal epilepsies: the role of the basal ganglia. NeuroImage Clin. (2017) 14:28–36. 10.1016/jnicl201612014 28123951 PMC5222946

[18] Wieser HG Blume WT Fish D Goldensohn E Hufnagel A King D. ILAE Commission Report. Proposal for a new classification of outcome with respect to epileptic seizures following epilepsy surgery. Epilepsia. (2001) 42:282–6. 10.1046/j1528-115720014220282x 11240604

[19] Gonzalez-Escamilla G Lange C Teipel S Buchert R Grothe MJ. PETPVE12: an SPM toolbox for partial volume effects correction in brain PET - application to amyloid imaging with AV45-PET. NeuroImage. (2017) 147:669–77. 10.1016/jneuroimage201612077 28039094

[20] Ashburner J. A fast diffeomorphic image registration algorithm. NeuroImage. (2007) 38:95–113. 10.1016/jneuroimage200707007 17761438

[21] Rousset OG Collins DL Rahmim A Wong DF. Design and implementation of an automated partial volume correction in PET: application to dopamine receptor quantification in the normal human striatum. J Nucl Med.(2008) 49:1097–106. 10.2967/jnumed107048330 18552147 PMC3104499

[22] Rubinov M Sporns O. Complex network measures of brain connectivity: uses and interpretations. NeuroImage. (2010) 52:1059–69. 10.1016/jneuroimage200910003 19819337

[23] Wang J Wang X Xia M Liao X Evans A He Y GRETNA: a graph theoretical network analysis toolbox for imaging connectomics. Front Hum Neurosci. (2015) 9:386 10.3389/fnhum201500386 26175682 PMC4485071

[24] Milo R Shen-Orr S Itzkovitz S Kashtan N Chklovskii D Alon U. Network motifs: simple building blocks of complex networks. Science. (2002) 298:824–7. 10.1126/science2985594824 12399590

[25] Latora V Marchiori M. Efficient behavior of small-world networks. Phys Rev Lett. (2001) 87:198701. 10.1103/PhysRevLett87198701 11690461

[26] Lamarche F Job AS Deman P Bhattacharjee M Hoffmann D Gallazzini-Crépin C. Correlation of FDG-PET hypometabolism and SEEG epileptogenicity mapping in patients with drug-resistant focal epilepsy. Epilepsia. (2016) 57:2045–55. 10.1111/epi13592 27861778 PMC5214566

[27] Almashaikhi T Rheims S Jung J Ostrowsky-Coste K Montavont A De Bellescize J. Functional connectivity of insular efferences. Hum Brain Mapp. (2014) 35:5279–94. 10.1002/hbm22549 24839121 PMC6869741

[28] Bernhardt BC Worsley KJ Besson P Concha L Lerch JP Evans AC. Mapping limbic network organization in temporal lobe epilepsy using morphometric correlations: insights on the relation between mesiotemporal connectivity and cortical atrophy. NeuroImage. (2008) 42:515–24. 10.1016/jneuroimage200804261 18554926

[29] Wang H David O Zhou W Wang L Zhang B Song X. Distinctive epileptogenic networks for parietal operculum seizures. Epilepsy Behav. (2019) 91:59–67. 10.1016/jyebeh201808031 30269938

[30] Wang X Hu W McGonigal A Zhang C Sang L Zhao B. Electroclinical features of insulo-opercular epilepsy: an SEEG and PET study. Ann Clin Transl Neurol. (2019) 6:1165–77. 10.1002/acn3789 31353858 PMC6649538

[31] Bartolomei F Gavaret M Hewett R Valton L Aubert S Régis J. Neural networks underlying parietal lobe seizures: a quantified study from intracerebral recordings. Epilepsy Res. (2011) 93:164–76. 10.1016/jeplepsyres201012005 21227653

[32] Caspers S Eickhoff SB Geyer S Scheperjans F Mohlberg H Zilles K. The human inferior parietal lobule in stereotaxic space. Brain Struct Funct. (2008) 212:481–95. 10.1007/s00429-008-0195-z 18651173

[33] Rushworth MF Behrens TE Johansen-Berg H. Connection patterns distinguish 3 regions of human parietal cortex. Cereb Cortex. (2006) 16:1418–30. 10.1093/cercor/bhj079 16306320

[34] Owen AM Coleman MR Boly M Davis MH Laureys S Pickard JD. Detecting awareness in the vegetative state. Science. (2006) 313:1402. 10.1126/science1130197 16959998

[35] McDonald CR Hagler DJ Ahmadi ME Tecoma E Iragui V Dale AM. Subcortical and cerebellar atrophy in mesial temporal lobe epilepsy revealed by automatic segmentation. Epilepsy Res. (2008) 79:130–8. 10.1016/jeplepsyres200801006 18359198 PMC2412955

[36] Englot DJ Konrad PE Morgan VL. Regional and global connectivity disturbances in focal epilepsy, related neurocognitive sequelae, and potential mechanistic underpinnings. Epilepsia. (2016) 57:1546–57. 10.1111/epi13510 27554793 PMC5056148

[37] Bullmore E Sporns O. Complex brain networks: graph theoretical analysis of structural and functional systems. Nat Rev Neurosci. (2009) 10:186–98. 10.1038/nrn2575 19190637

[38] Haneef Z Chiang S. Clinical correlates of graph theory findings in temporal lobe epilepsy. Seizure. (2014) 23:809–18. 10.1016/jseizure201407004 25127370 PMC4281255

