# Supplementary material for: Aberrant Metabolic Patterns Networks in Insular Epilepsy
Source: Front Neurol. 2020 Dec 23;11:605256. doi: 10.3389/fneur.2020.605256 (PMC7786135; doi:10.3389/fneur.2020.605256)
Supplement: Supplementary file 1 [file Table_1.DOCX]

**Aberrant** **metabolic patterns networks in insular epilepsy**

**Supplement Materials**

**Network metrics based on Brain Connectome Toolbox [1]**

**1. Global network metrics**

**(1) Small-world property**: Small-world networks were formally defined as networks that were simultaneously highly segregated and integrated.

$$\sigma=\frac{C/Cr}{L/Lr}$$

*C* and *Cr*: clustering coefficient of tested and random network. *L* and *Lr*: path length of tested and random network. Small-world scalar *σ* > 1, both *γ* = *C*/*Cr* > 1 and λ = *L*/*Lr* ∼ 1 are also required.

**(2) Global efficiency (binary, weighted):** The global efficiency is the average of inverse shortest path length, and is inversely related to the characteristic path length.

$$E^{w} = \frac{1}{n}\sum_{i\in N} \frac{\sum_{j\in N,j\neq i} {(w_{ij}^{w})}^{-1}}{n-1}$$

*N* is the set of all nodes in the network, and *n* is the number of nodes. *(i, j)* is a link between nodes *i* and *j*, *(i, j ∈ N)*. *d* is shortest path length (distance), between nodes *i* and *j*.

**2. Nodal network metrics**

**(1) Clustering coefficient (weighted, undirected):** The weighted clustering coefficient is the average "intensity" (geometric mean) of all triangles associated with each node.

$$C^{w}= \frac{1}{n}\sum_{i\in N} \frac{2t_{i}^{w}}{k_{i}(k_{i}-1)}$$

*N* is the set of all nodes in the network, and *n* is the number of nodes. *t_i_* is the number of triangles around a node *i*. *k_i_* is the degree of a node *i*.

**(2) Degree Centrality:** The degree centrality is a statistic that quantifies the nodal degree for a given node reflects its information communication ability in the functional network.

$$DC \left( i \right) = \sum_{j=1}^{l} a_{ij}$$

l is number of links. (i, j) is a link between nodes i and j, (i, j ∈ N).

**(3) Local efficiency (weighted, undirected):** The local efficiency is the global efficiency computed on the neighbourhood of the node, and is related to the clustering coefficient.

$$E_{loc}^{w} = \frac{1}{2}\sum_{i\in N} \frac{\sum_{j,h\in N,j\neq i} {(w_{ij}w_{ih}{[d_{jh}^{w}(N_{i})]}^{-1})}^{1/3}}{k_{i}(k_{i}-1)}$$

*N* is the set of all nodes in the network. *(i, j)* is a link between nodes *i* and *j*, *(i, j ∈ N)*. *k_i_* is the degree of a node *i*.

**(4) Betweenness centrality (undirected):** Node betweenness centrality is the fraction of all shortest paths in the network that contain a given node.

$$b_{i} = \frac{1}{(n -1)(n -2)}\sum_{\begin{aligned} h,j\in N \\ h\neq j,h\neq i,j\neq i \end{aligned}} \frac{{\rho_{hj}}^{(i)}}{\rho_{hj}}$$

*N* is the set of all nodes in the network, and *n* is the number of nodes. *(i, j, h)* is a link between nodes *i, j* and *h*, *(i, j, h ∈ N)*. *ρ_hj_* is the number of shortest paths between *h* and *j*, and *ρ_hj_ ^(i)^* is the number of shortest paths between *h* and *j* that pass-through *i*.

**Table S1.** Neuroimaging information of individuals with insular epilepsy.

| Patient | Co-registration of MRI/PET | Co-registration of MRI and post-surgical CT |
| --- | --- | --- |
| 1 | 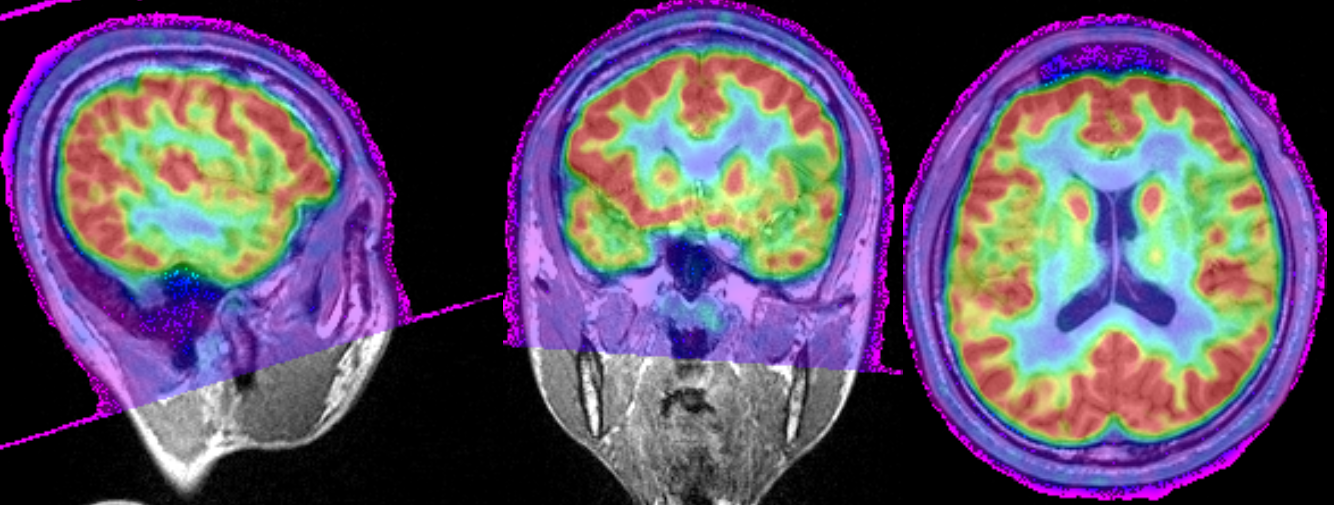 | 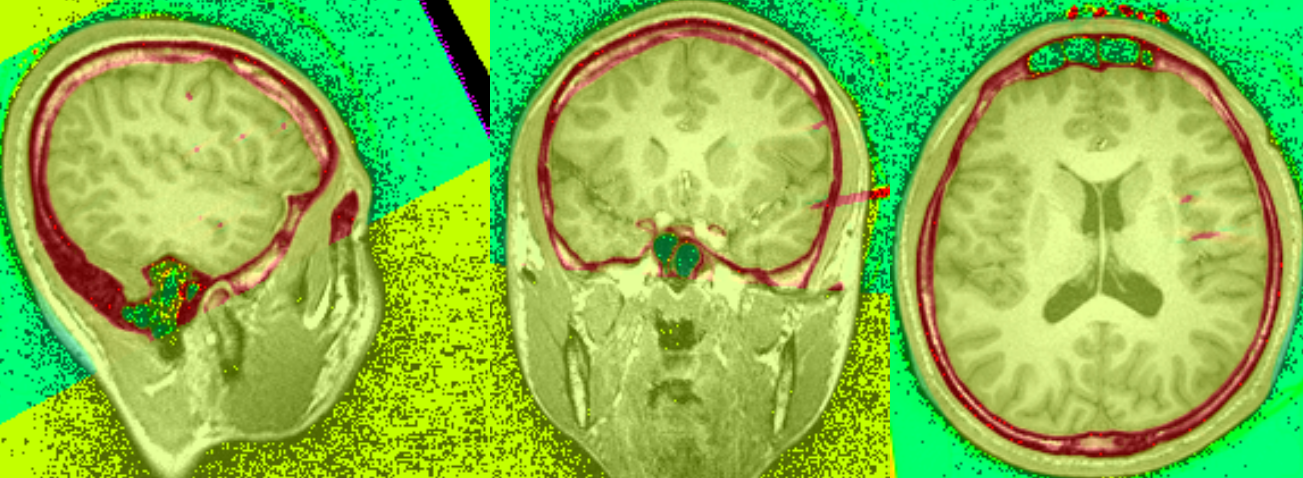 |
| 2 | 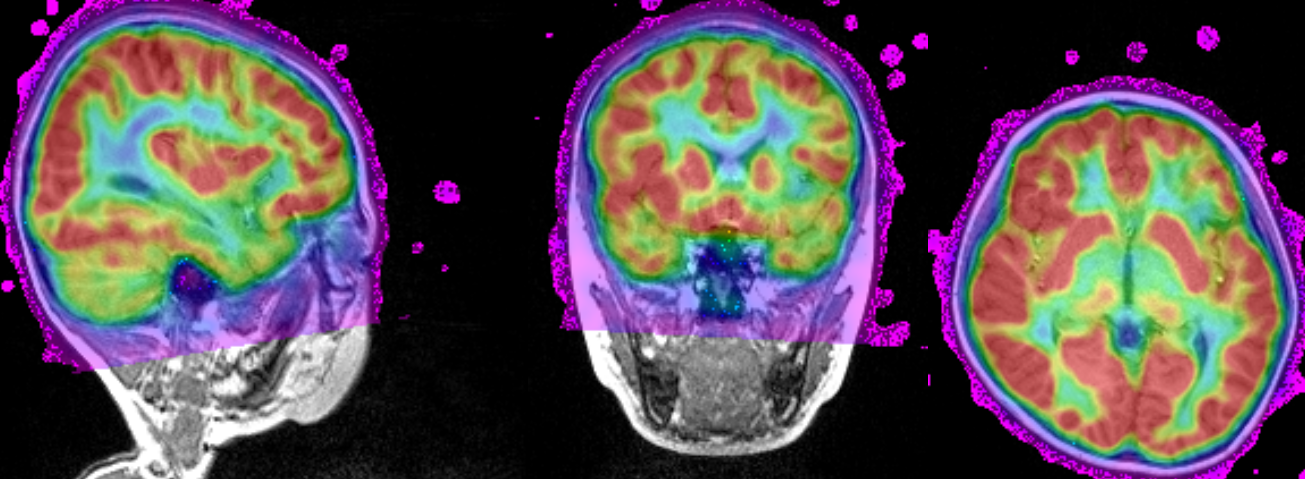 | 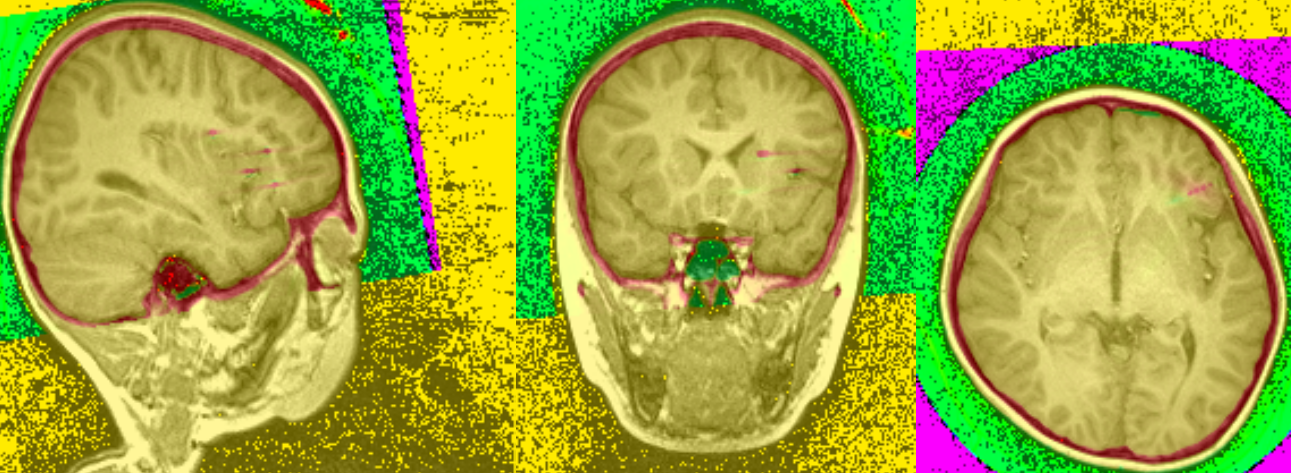 |
| 3 | 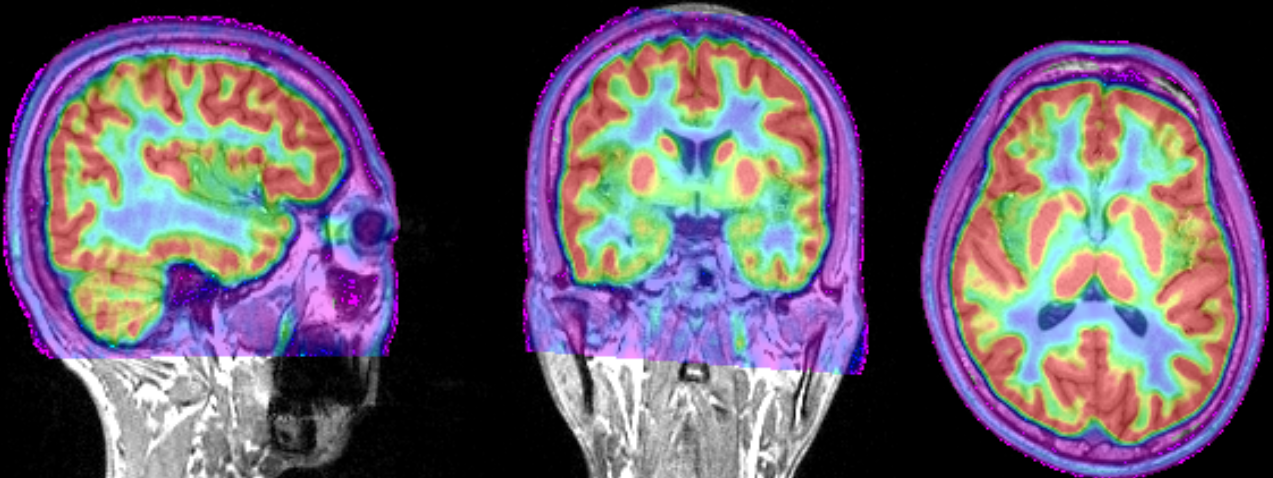 | 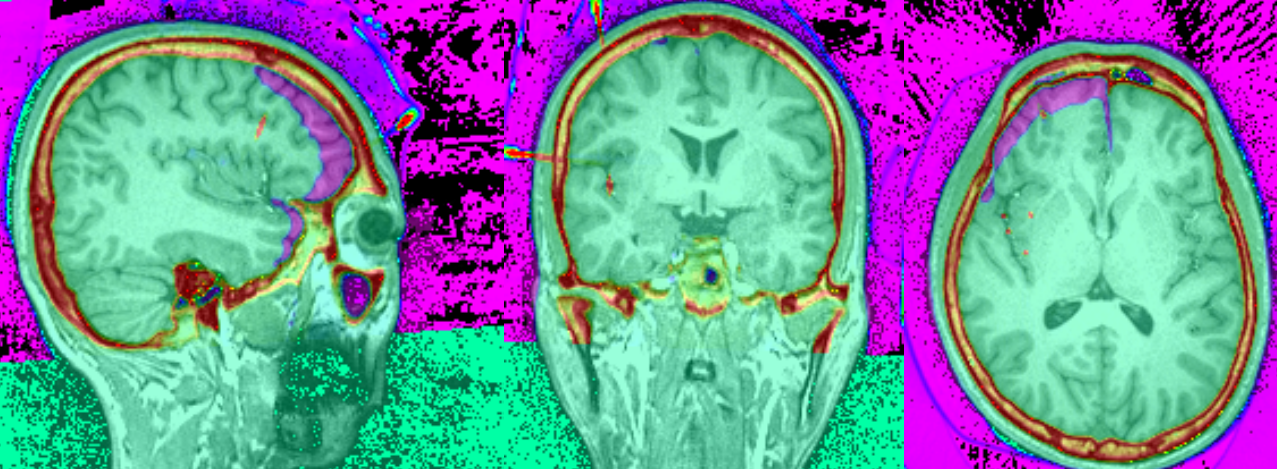 |
| 4 | 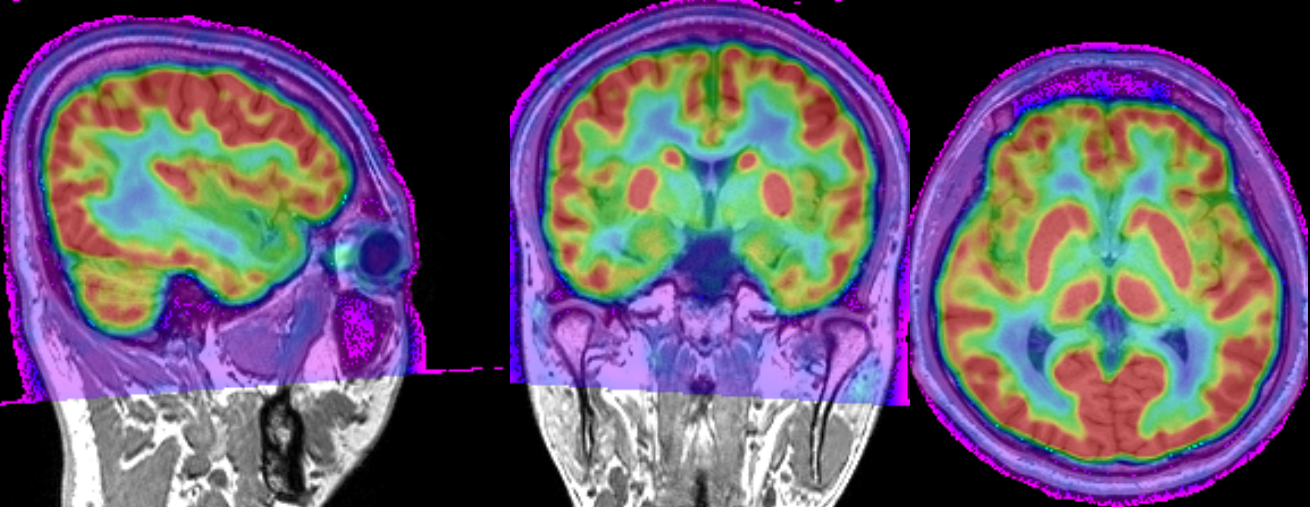 | 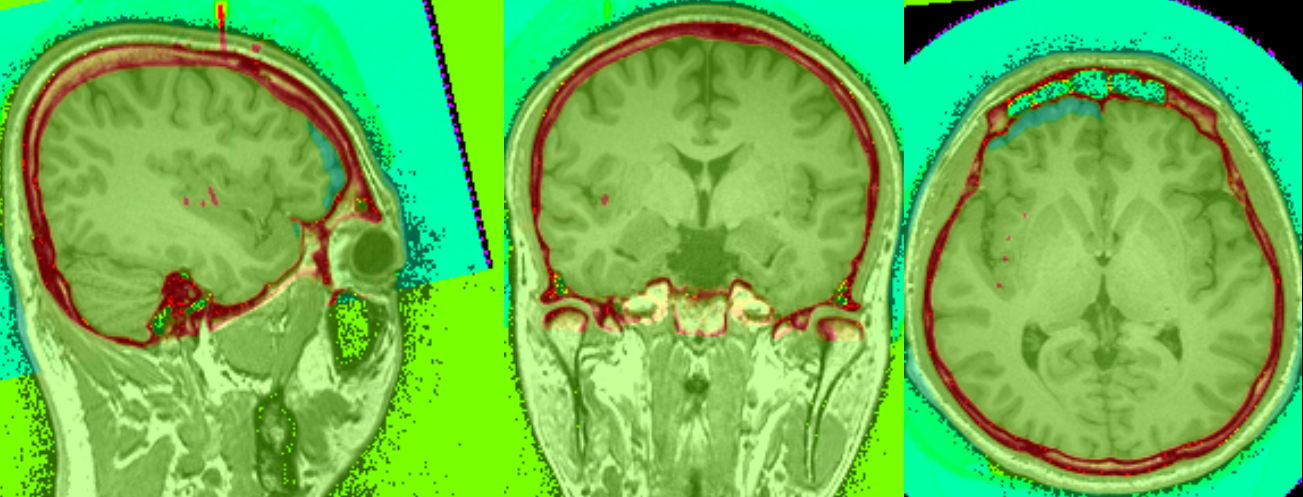 |
| 5 | 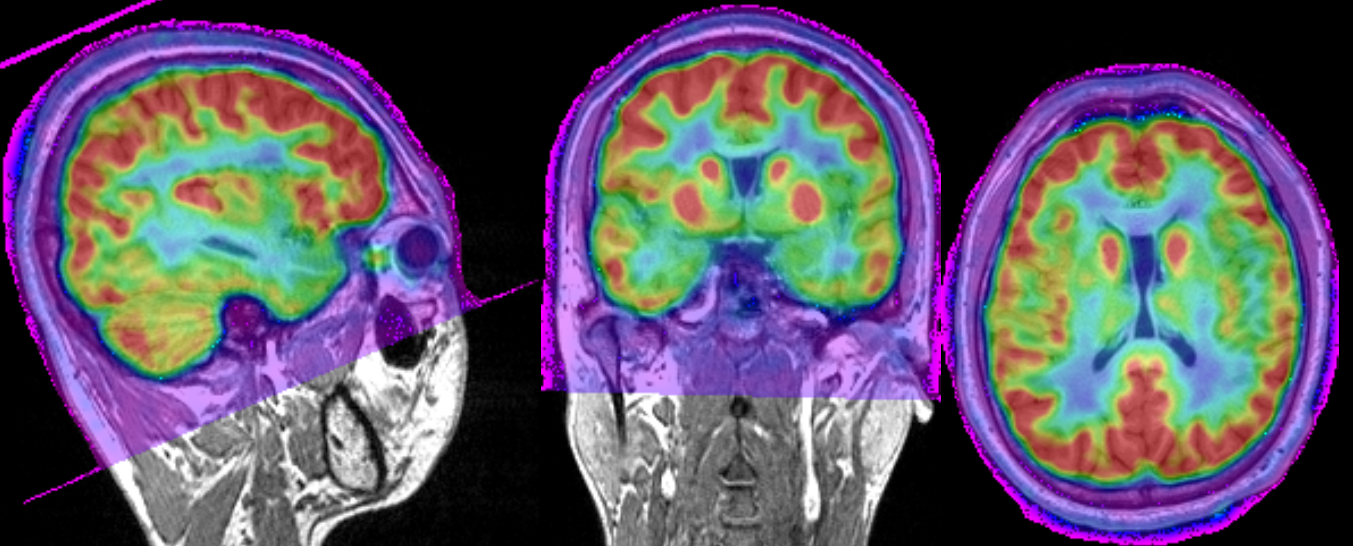 | 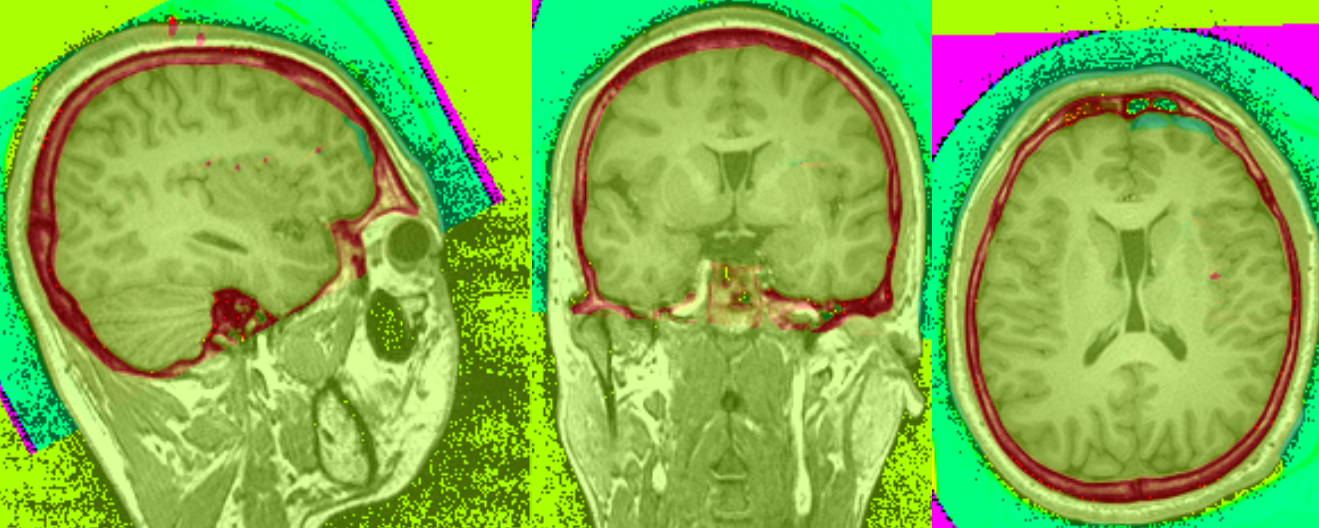 |
| 6 | 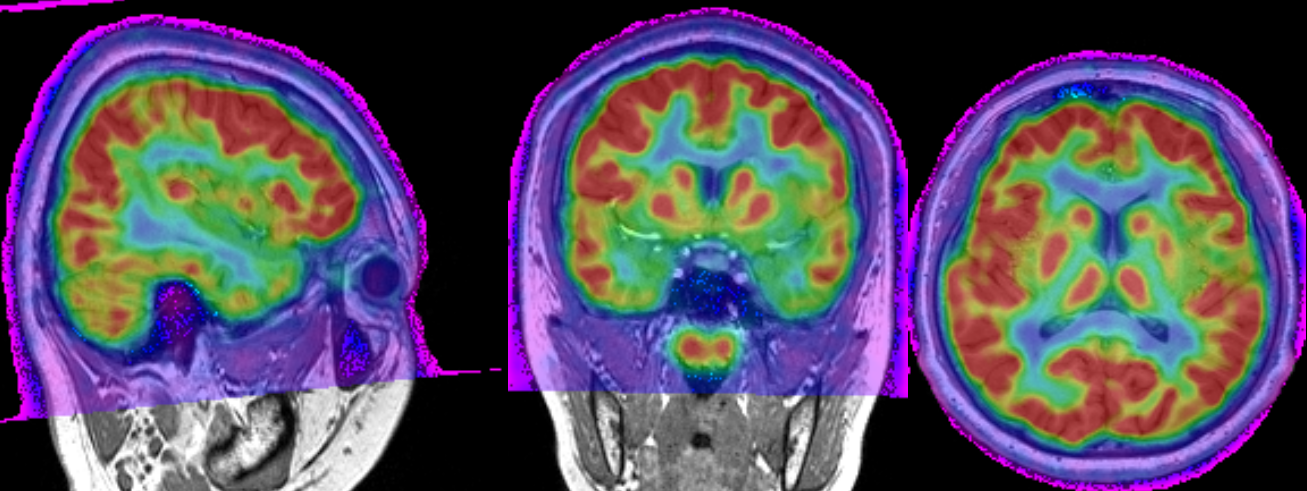 | 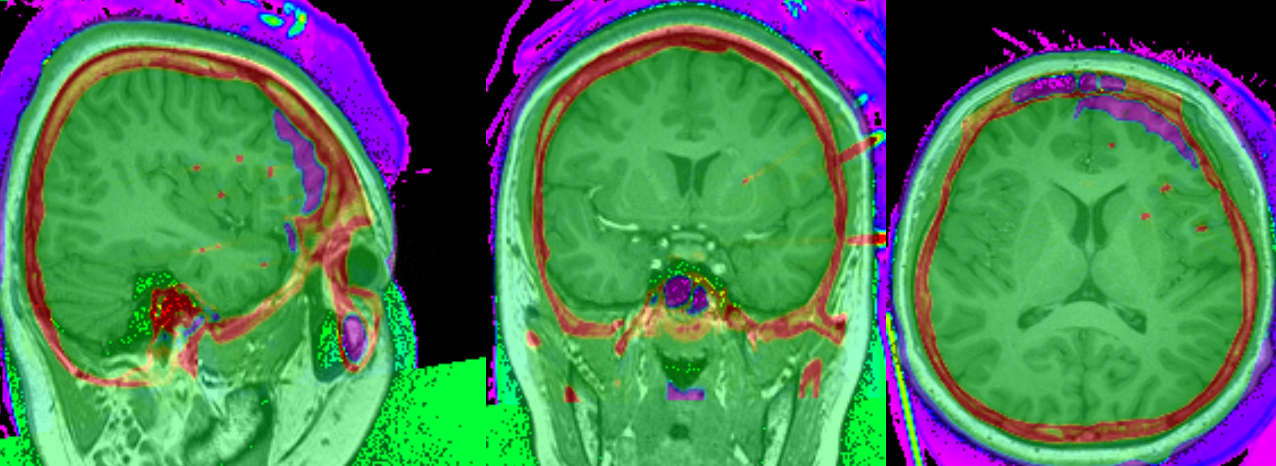 |
| 7 | 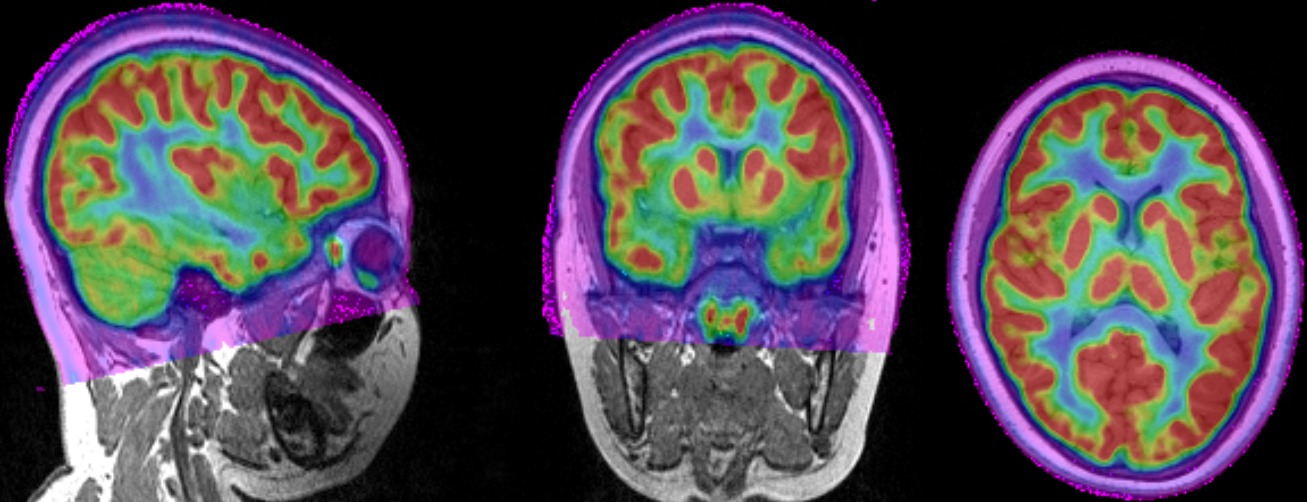 | 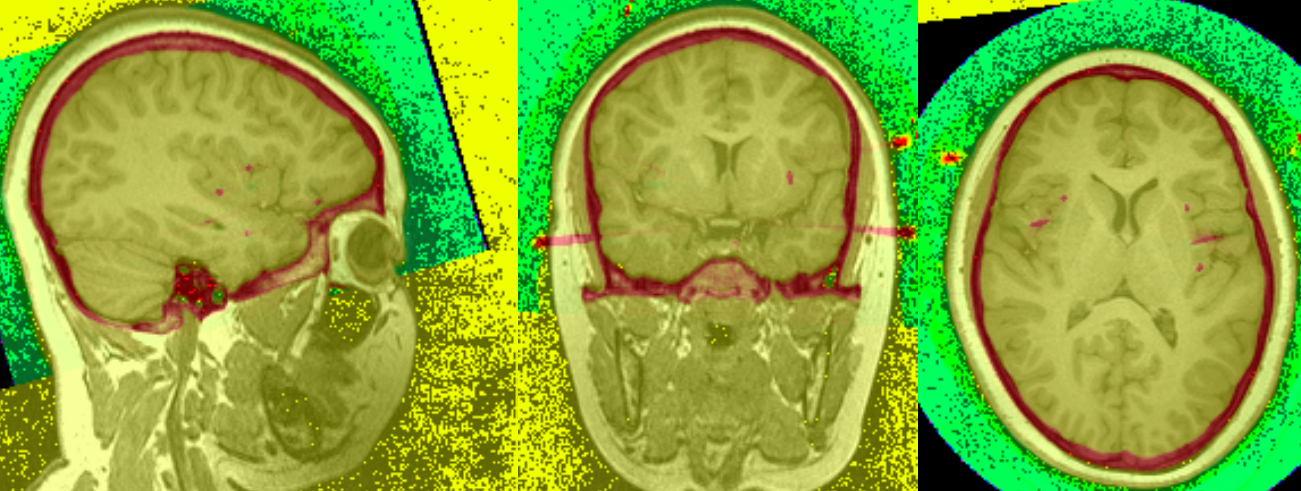 |
| 8 | 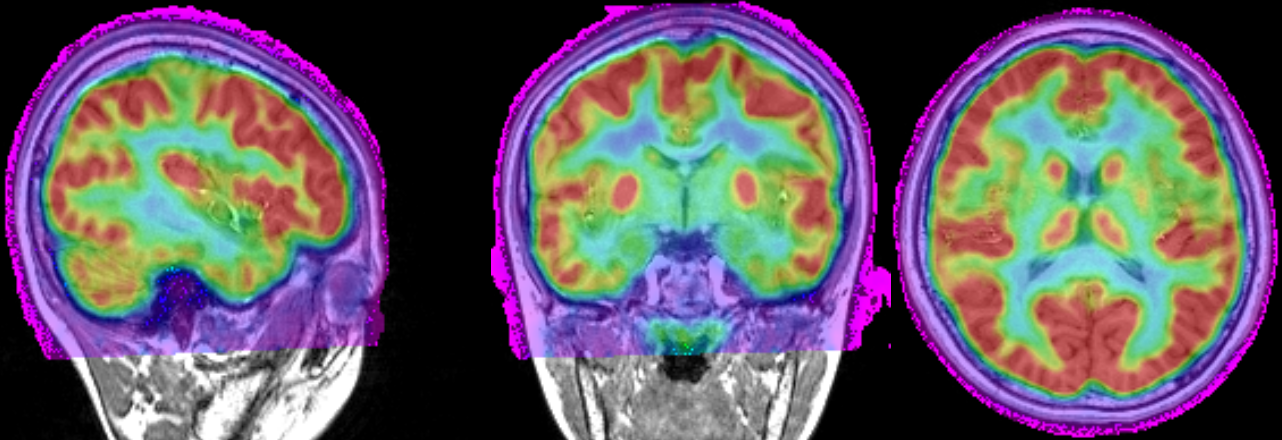 | 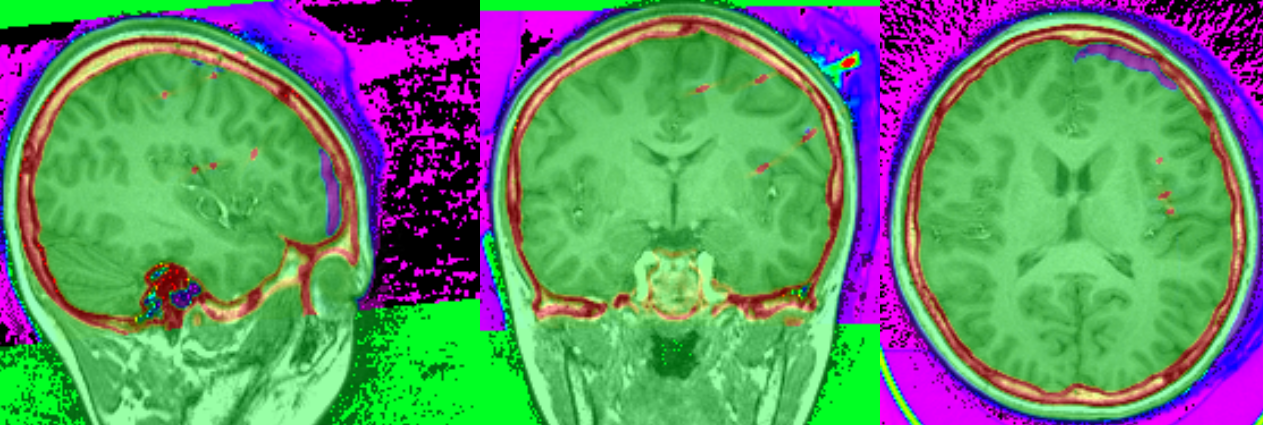 |
| 9 | 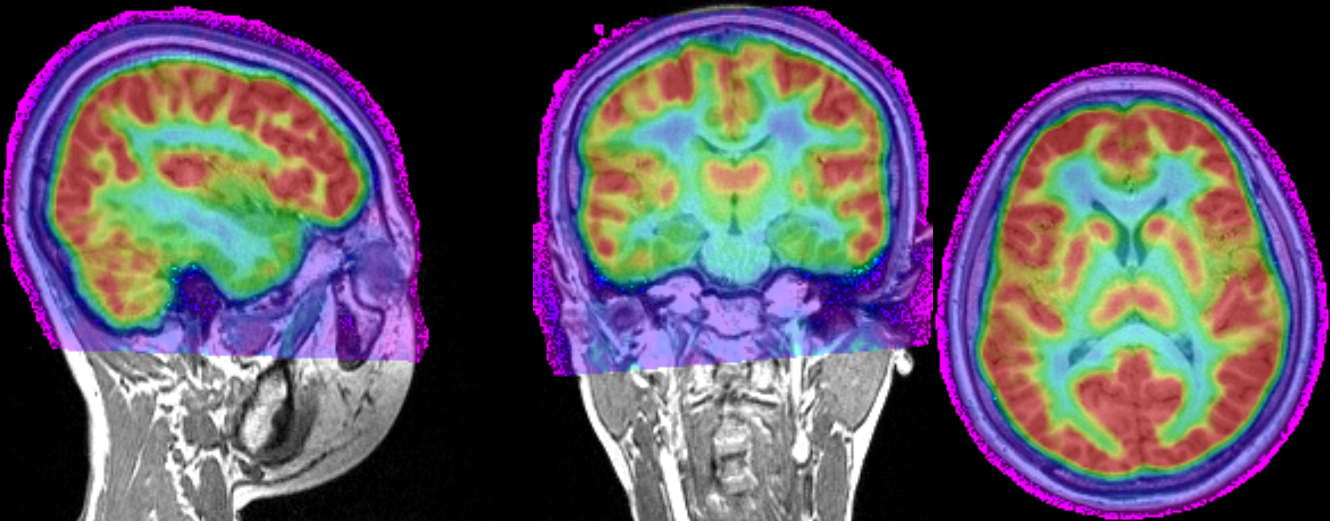 | 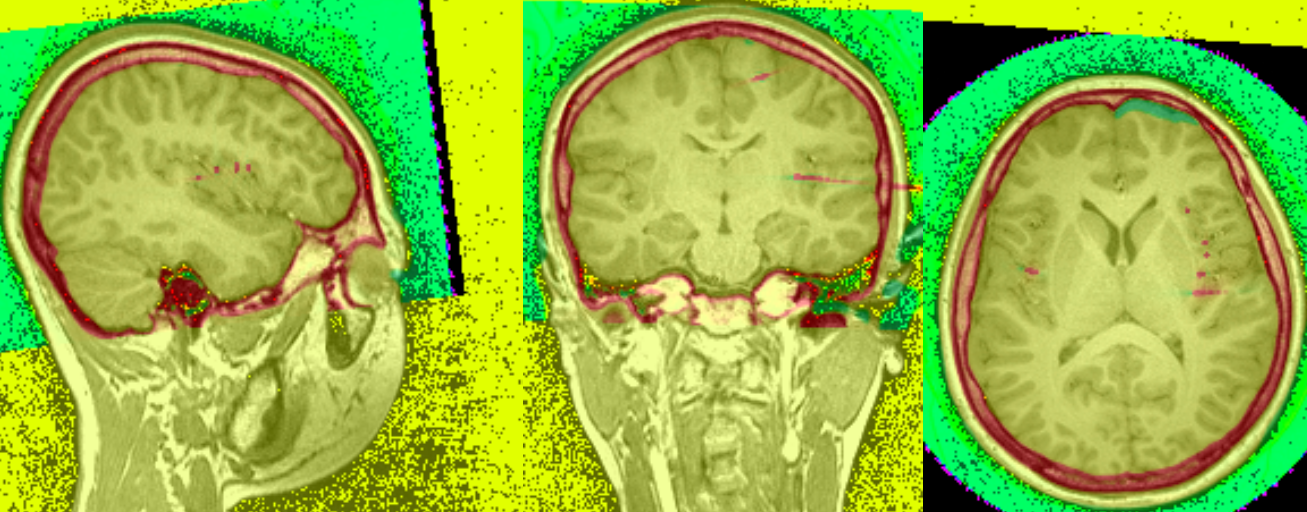 |
| 10 | 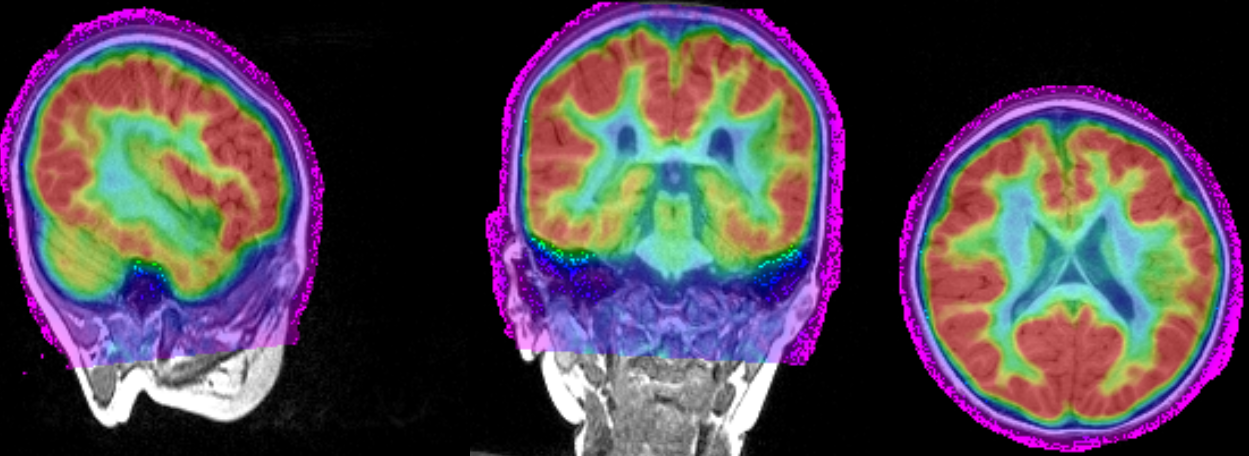 | 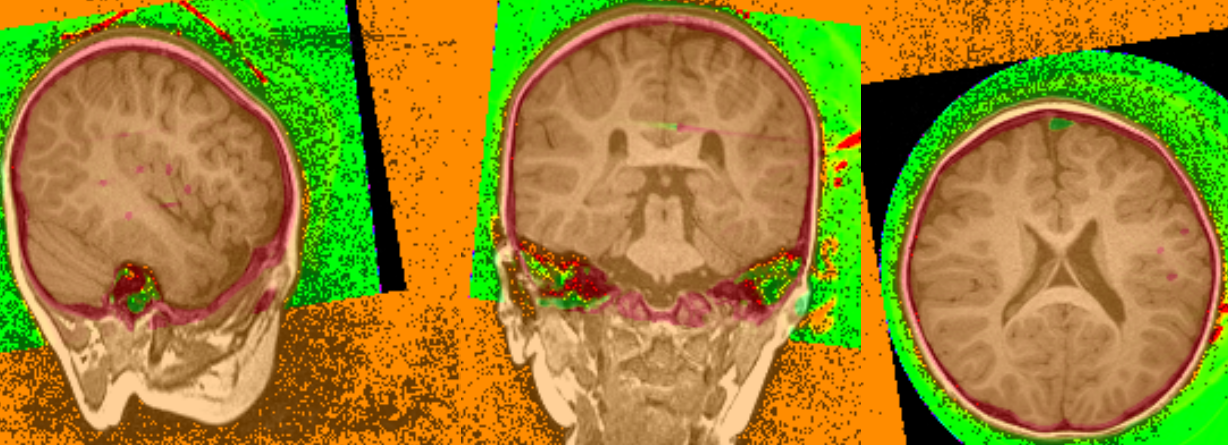 |
| 11 | 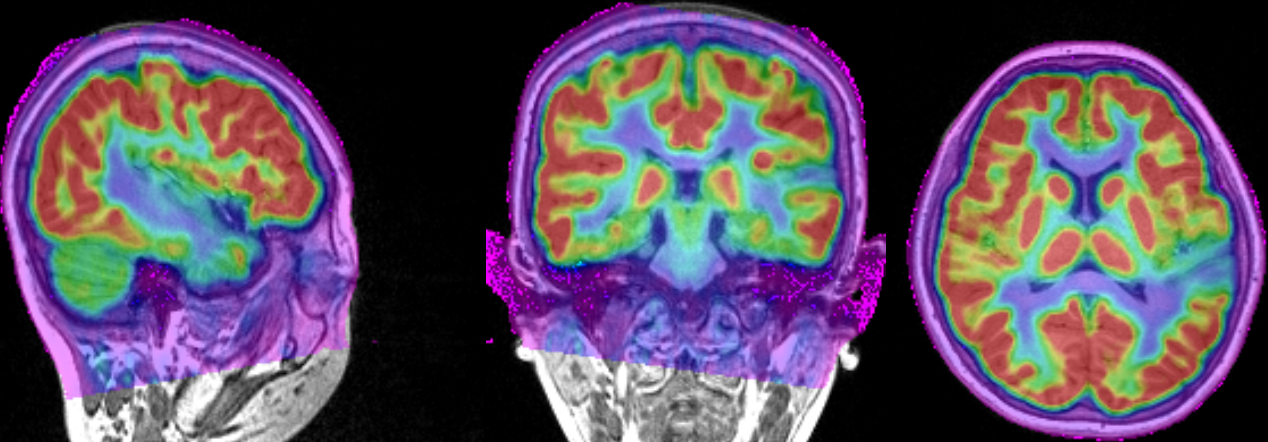 | 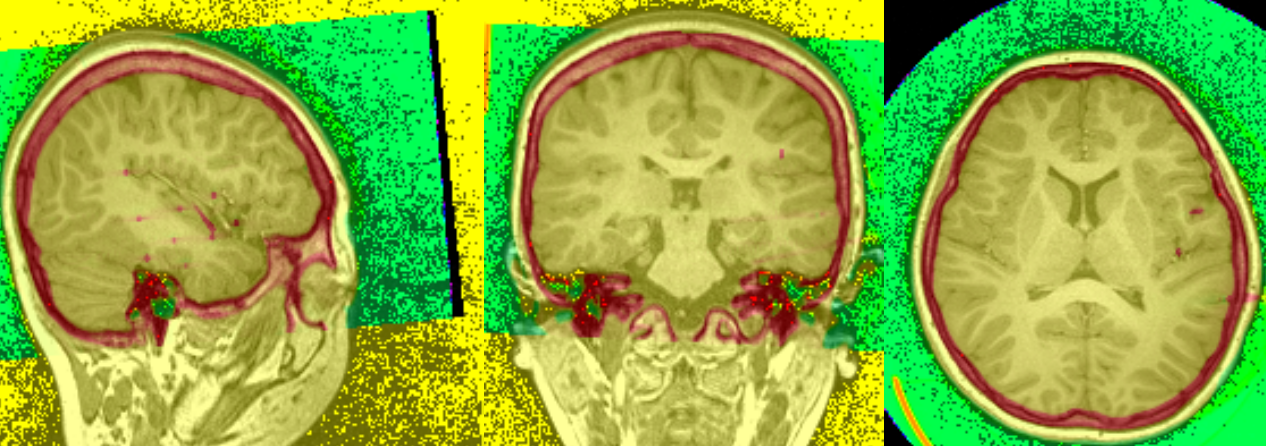 |
| 12 | 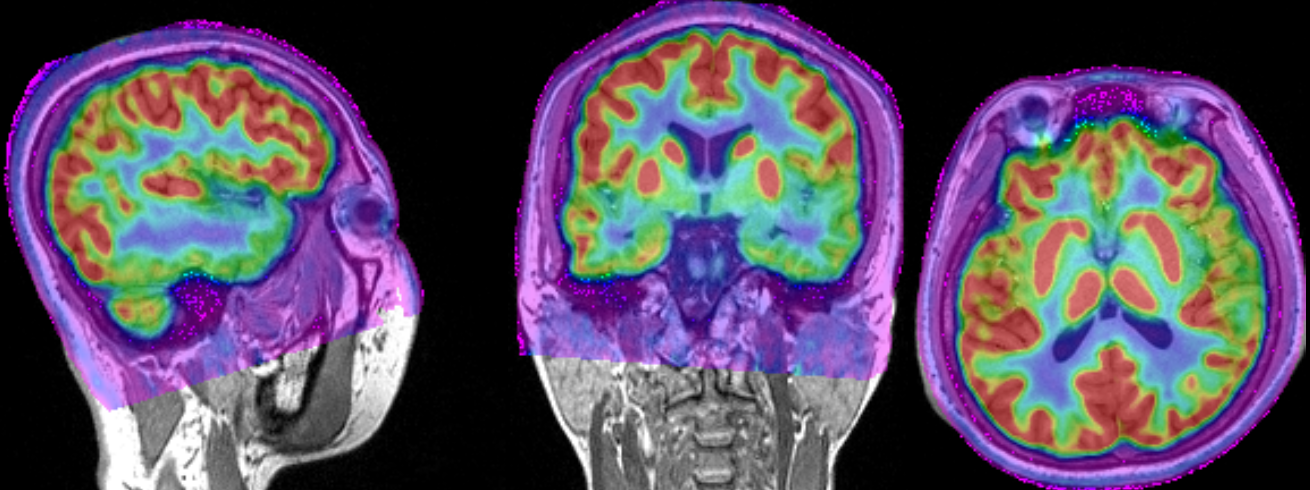 | 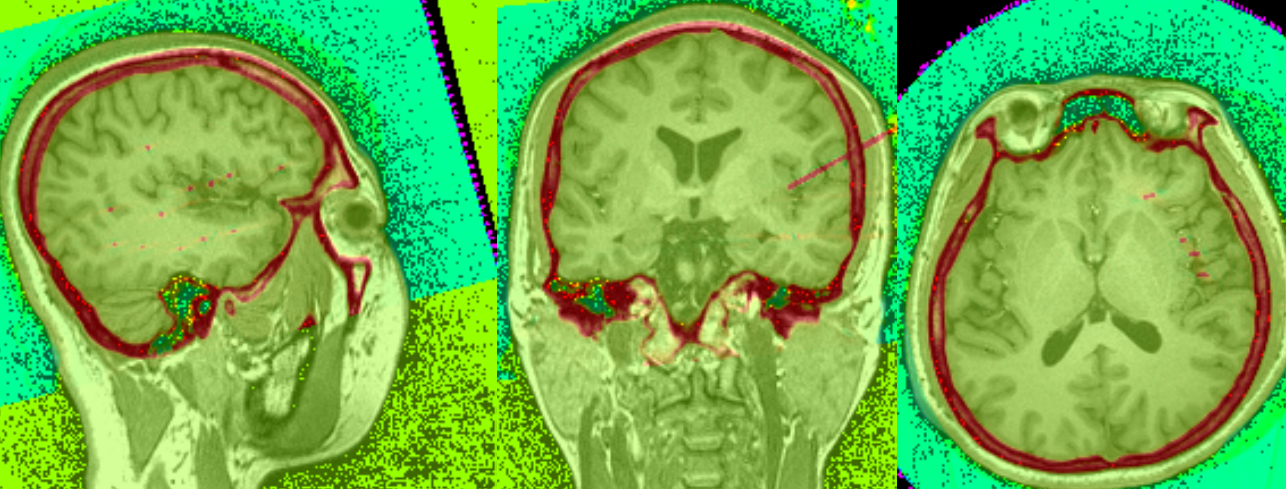 |
| 13 | 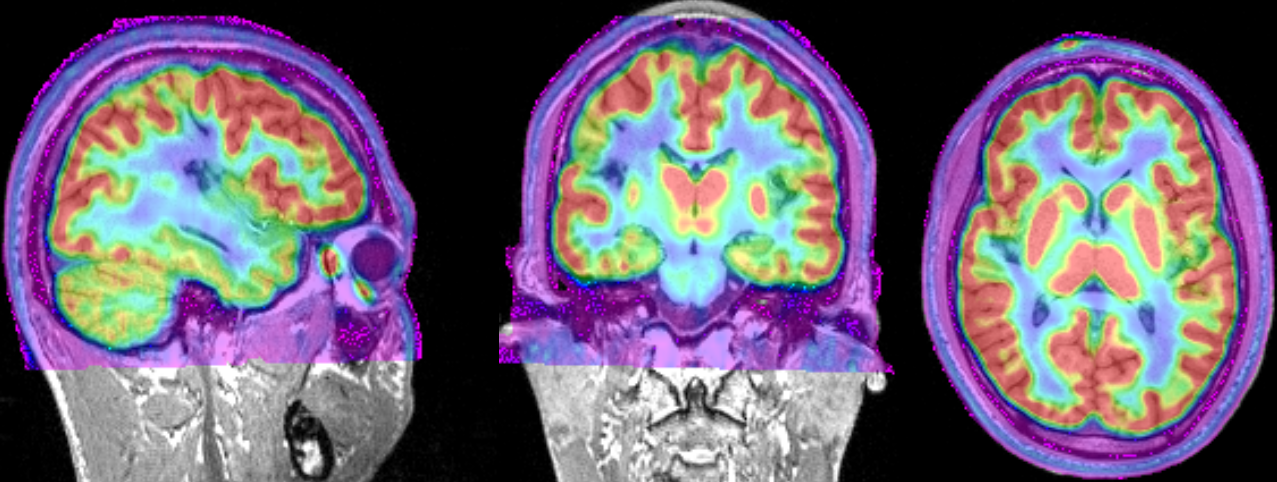 | 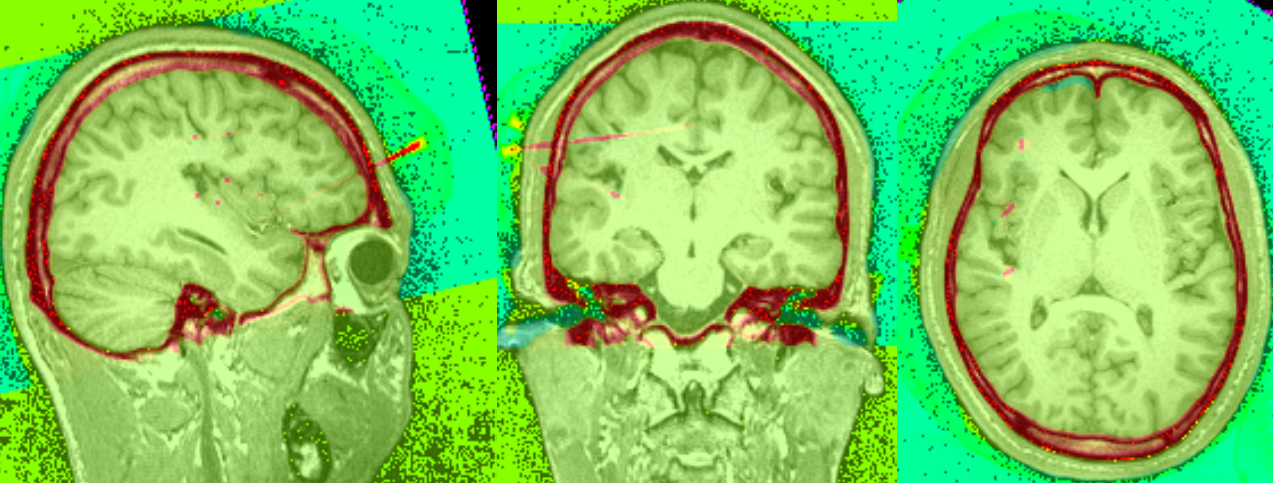 |
| 14 | 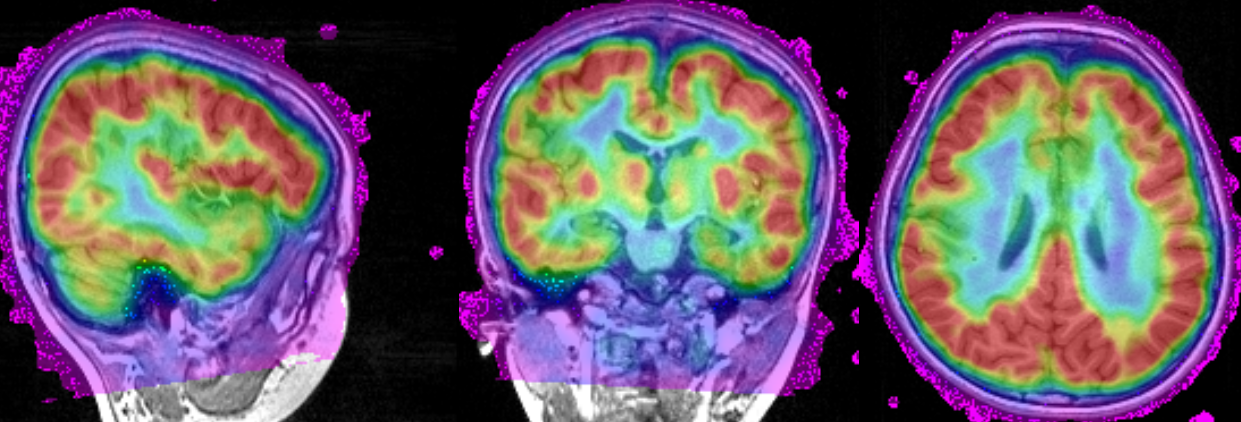 | 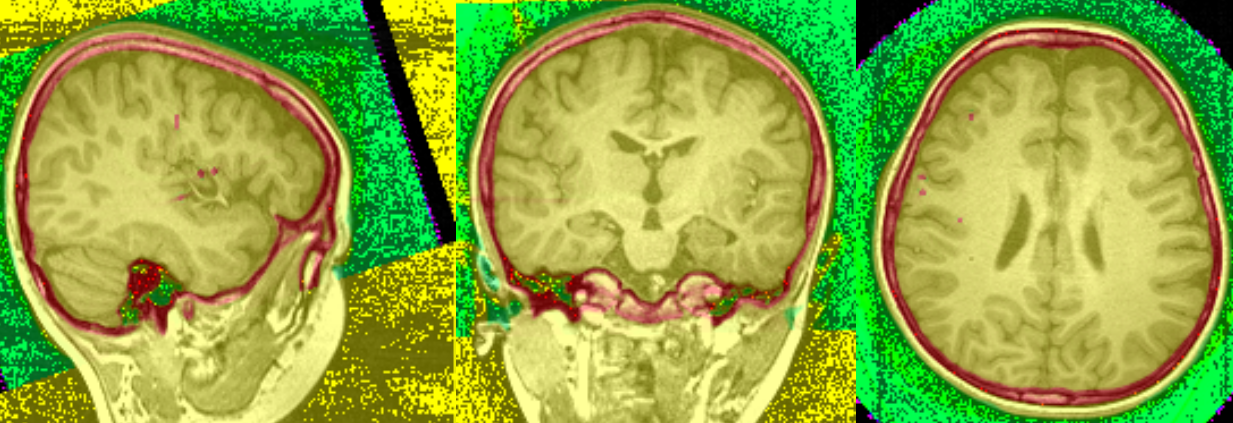 |
| 15 | 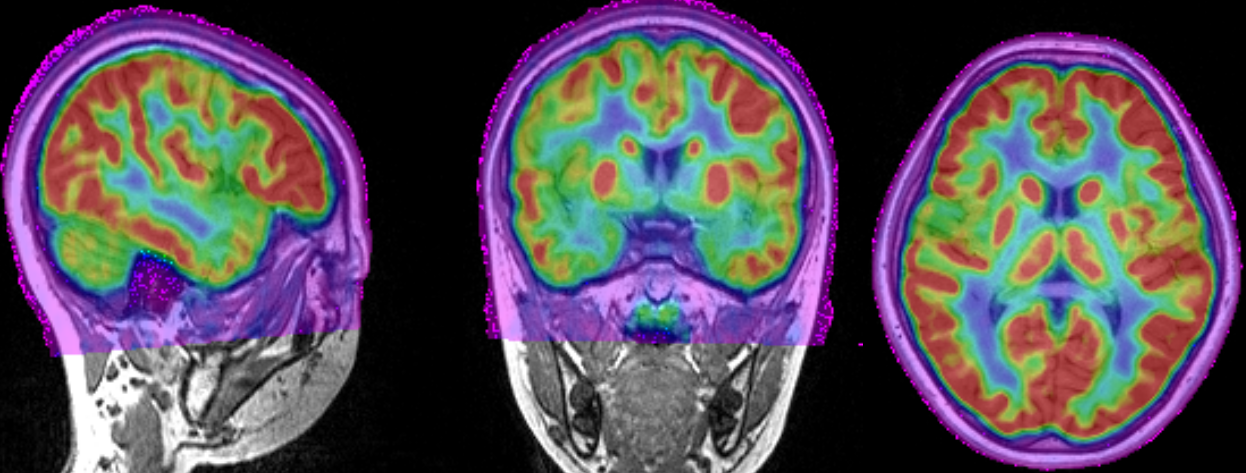 | 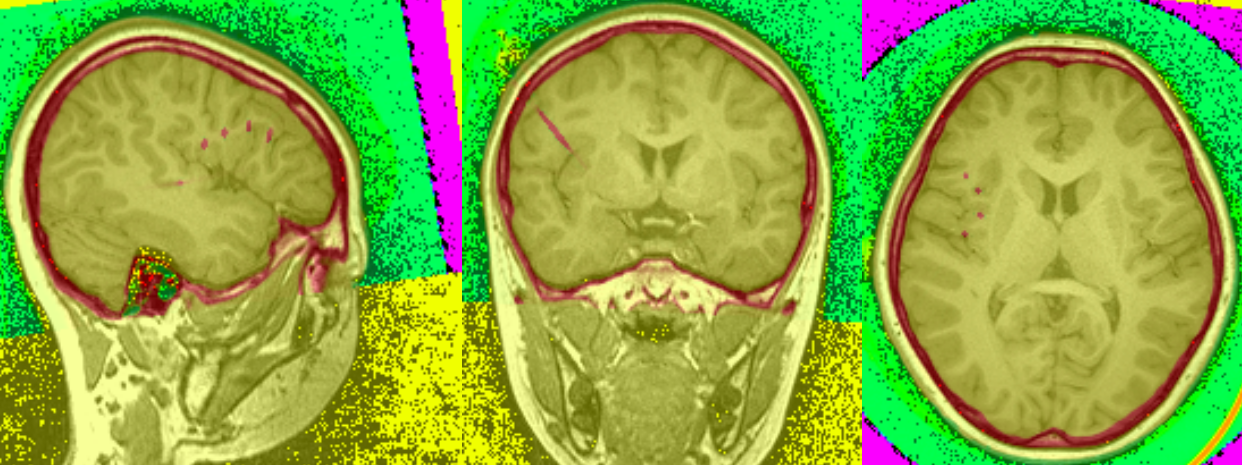 |
| 16 | 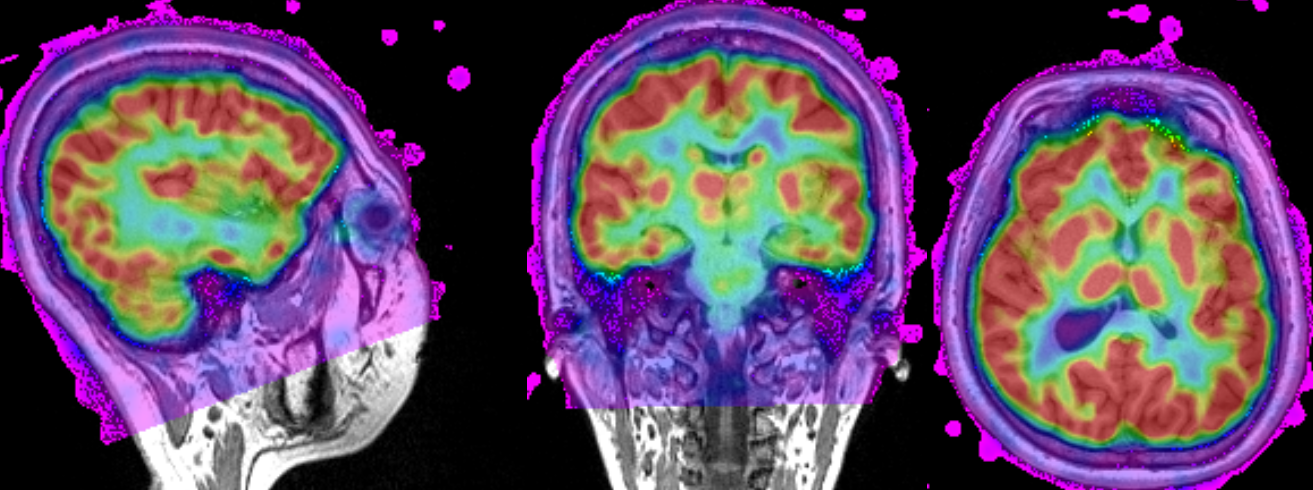 | 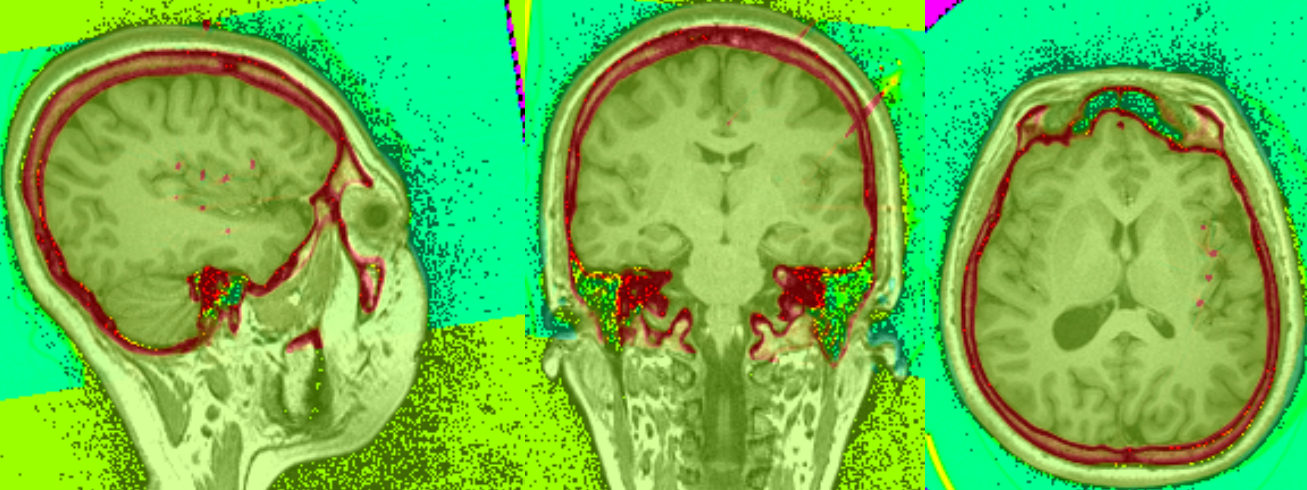 |
| 17 | 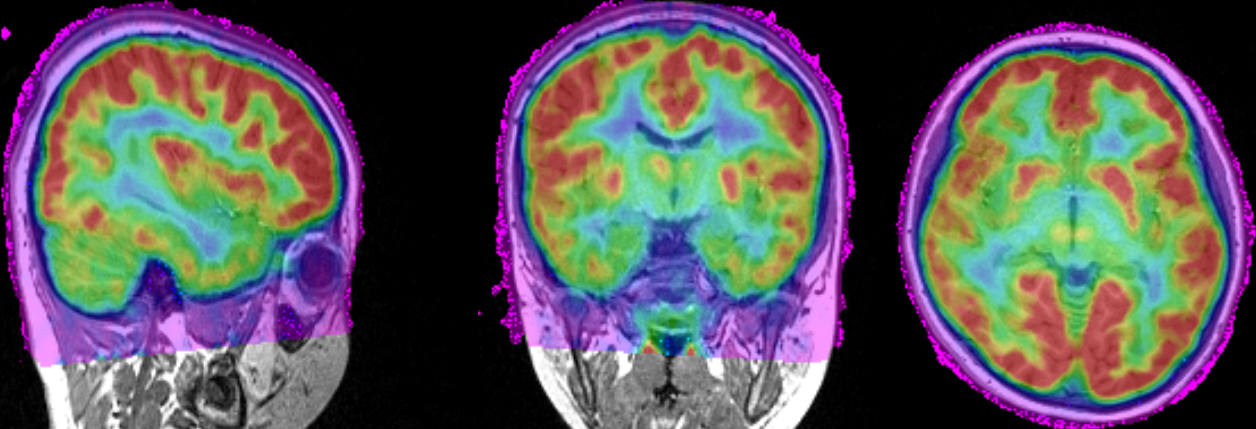 | 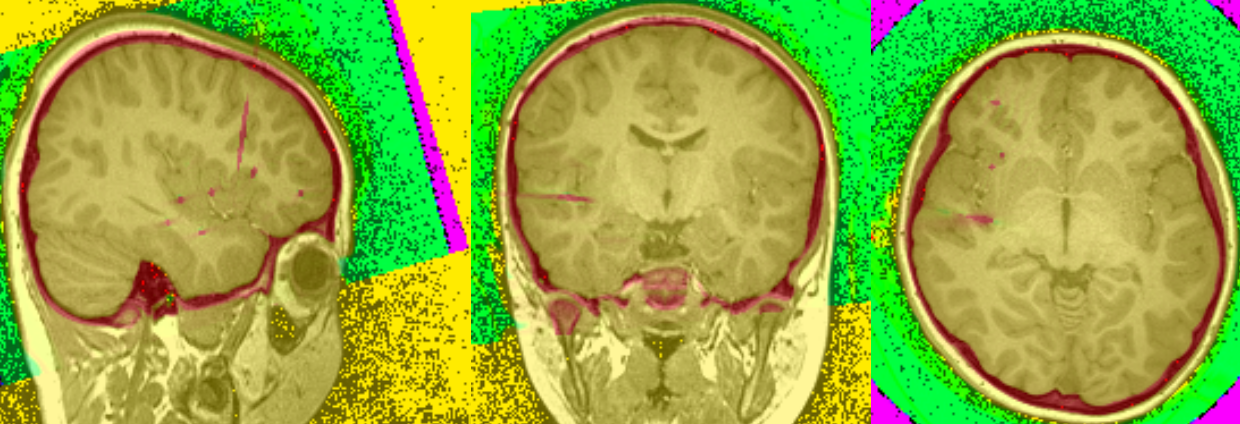 |

**Table S2.** Abbreviations and full name of included brain structures from AAL and AAN atlas [2]

| Abbreviations | Full name | Abbreviations | Full name |
| --- | --- | --- | --- |
| 39 neocortical structures | | Precuneus | Precuneus |
| Insula | Insula | Fusiform | Fusiform gyrus |
| Precentral | Precentral gyrus | ParaHippocampal | Parahippocampal gyrus |
| Frontal Sup | Superior frontal gyrus, dorsolateral | Cingulum Post | Posterior cingulate gyrus |
| Frontal Sup Orb | Superior frontal gyrus, orbital part | Cingulum Mid | Median cingulate and paracingulate gyri |
| Frontal Mid | Middle frontal gyrus | Cingulum Ant | Anterior cingulate and paracingulate gyri |
| Frontal Mid Orb | Middle frontal gyrus, orbital part | Rectus | Gyrus rectus |
| Frontal Inf Oper | Inferior frontal gyrus, opercular part | Frontal Med Orb | Superior frontal gyrus, orbital part |
| Frontal Inf Tri | Inferior frontal gyrus, triangular part | Frontal Sup Medial | Superior frontal gyrus, medial orbital |
| Frontal Inf Orb | Superior frontal gyrus, medial orbital | Olfactory | Olfactory cortex |
| Rolandic Oper | Rolandic operculum | Supp Motor Area | Supplementary motor area |
| Heschl | Heschl gyrus |  |  |
| Temporal Sup | Superior temporal gyrus | 6 subcortical structures | |
| Temporal Pole Sup | Temporal pole: superior temporal gyrus | Hippocampus | Hippocampus |
| Temporal Mid | Middle temporal gyrus | Amygdala | Amygdala |
| Temporal Pole Mid | Temporal pole: middle temporal gyrus | Caudate | Caudate nucleus |
| Temporal Inf | Inferior temporal gyrus | Putamen | Putamen |
| Postcentral | Postcentral gyrus | Pallidum | Pallidum |
| Parietal Sup | Superior parietal gyrus | Thalamus | Thalamus |
| Parietal Inf | Inferior parietal gyrus |  |  |
| SupraMarginal | Supramarginal gyrus | 8 brainstem structures | |
| Angular | Angular gyrus | DR | Dorsal raphé nucleus |
| Occipital Sup | Superior occipital gyrus | LC | Locus coeruleus |
| Occipital Mid | Middle occipital gyrus | MR | Median raphé nucleus |
| Occipital Inf | Inferior occipital gyrus | PAG | Periaqueductal grey |
| Lingual | Lingual gyrus | PBC | Parabrachial complex |
| Cuneus | Cuneus | PO | Pontis nucleus oralis |
| Calcarine | Calcarine fissure and surrounding cortex | PPN | Pedunculopontine nucleus |
| Paracentral Lobule | Paracentral lobule | VTA | Ventral tegmental area |


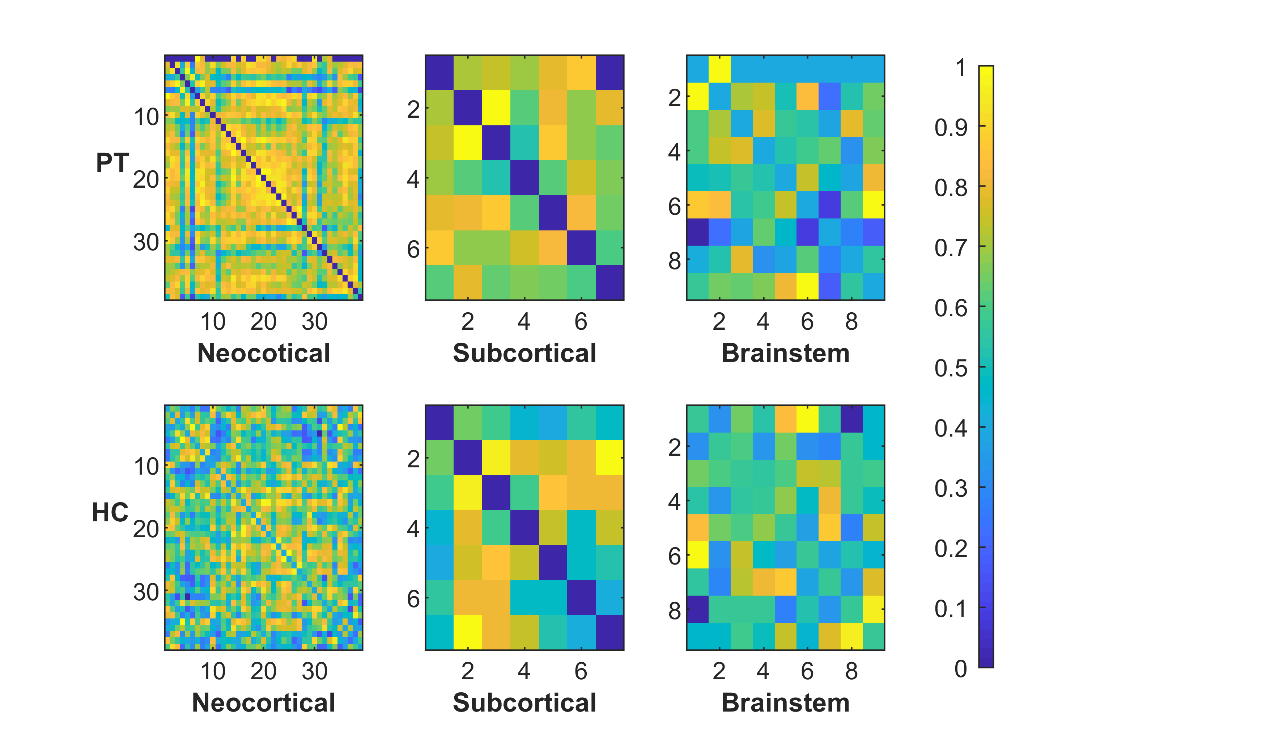


**Figure S1.** Insula-to-cortex partial correlation matrix for patients with insular epilepsy (upper panel) and healthy controls (lower panel).

**References**

1. M R, O S: **Complex network measures of brain connectivity: uses and interpretations**. *NeuroImage* 2010, **52**(3):1059-1069.

2. N T-M, B L, D P, F C, O E, N D, B M, M J: **Automated anatomical labeling of activations in SPM using a macroscopic anatomical parcellation of the MNI MRI single-subject brain**. *NeuroImage* 2002, **15**(1):273-289.
